# The key role of chirality and peripheral substitution in the columnar organization of bowl-shaped subphthalocyanines†

**DOI:** 10.1039/d4sc03976a

**Published:** 2024-07-29

**Authors:** Jorge Labella, Elisa López-Serrano, Daniel Aranda, María J. Mayoral, Enrique Ortí, Tomás Torres

**Affiliations:** a Department of Organic Chemistry, Universidad Autónoma de Madrid Campus de Cantoblanco, C/Francisco Tomás y Valiente 7 28049 Madrid Spain jorge.labella@uam.es tomas.torres@uam.es; b Instituto de Ciencia Molecular (ICMol), Universidad de Valencia 46980 Paterna Spain enrique.orti@uv.es; c Andalucía Tech, Facultad de Ciencias, Departamento de Química Física, Universidad de Málaga 29071 Málaga Spain; d Inorganic Chemistry Department, Universidad Complutense de Madrid 28040 Madrid Spain; e Institute for Advanced Research in Chemical Sciences (IAdChem), Universidad Autónoma de Madrid Madrid Spain; f IMDEA – Nanociencia C/Faraday 9, Campus de Cantoblanco 28049 Madrid Spain

## Abstract

The columnar arrangement of bowl-shaped aromatics is a promising strategy for producing high-performing semiconductors. However, the structural factors that dictate the self-assembly of these molecules remain poorly understood. Herein, we show how chirality and peripheral substitution affect the columnar assembly of subphthalocyanines (SubPcs) in solution. Both aspects are found to influence the structure, stability, and formation mechanism of the supramolecular polymer obtained. Whereas enantiopure tri-substituted SubPcs cooperatively polymerize into homochiral head-to-tail arrays, racemic mixtures socially self-sort, leading to heterochiral columnar polymers. In sharp contrast, hexa-substituted SubPcs polymerize following an isodesmic mechanism, producing highly robust columnar systems. As elucidated by molecular dynamics calculations, the conformational flexibility of these SubPcs, as well as the number of peripheral groups able to intermolecularly interact, underlie these significant differences. The results presented herein pave the way for the realistic application of bowl-shaped π-compounds.

## Abstract 2

This work describes the crucial effect that optical purity and substitution pattern exert on the formation mechanism and stability of columnar polymers formed from the self-assembly of bowl-shaped subphthalocyanines.

## Graphical abstract

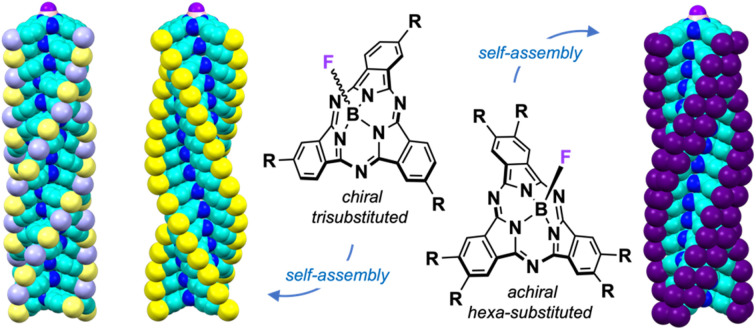

## Introduction

The supramolecular assembly of π-conjugated molecules into columnar architectures has become a powerful strategy to prepare soft and reconfigurable materials with intriguing charge transport capabilities.^1^ In this context, increasing complexity, but also a unique functional landscape, arises when molecules with a bowl-shaped structure are employed as building blocks.^2^ Bowl-shaped molecules exhibit a permanent dipole moment.^3^ Consequently, their columnar assembly develops polarization along the stacking axis, leading to unique properties of high technological value, such as ferroelectricity^4^ or the bulk-photovoltaic effect (BPVE).^5^ In addition, bowl-shaped compounds with a non-centrosymmetric structure can become intrinsically chiral through proper functionalization.^6^ These chiral building blocks can be self-assembled in enantiopure nanostructures opening up opportunities for exploitation in the rapidly growing field of chiral technologies.^7^ Nevertheless, the development of this kind of material is sluggish due to the challenge of finding concave π-scaffolds combining proper photophysical properties and a good ability to be organized columnarly in a controlled manner.

**Fig. 1:**
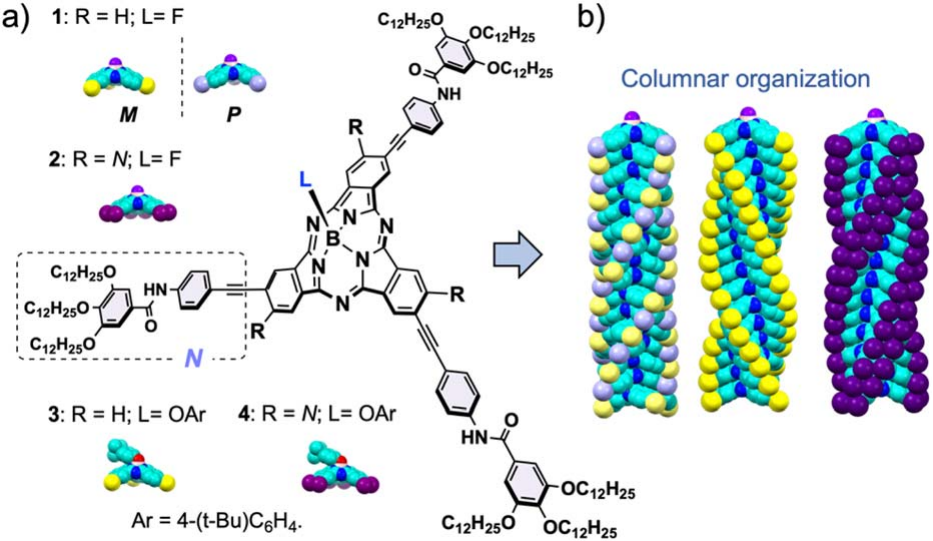
(a) Molecular structures of the SubPcs used as self-assembling units in this study. (b) 3D model of their columnar organization. Atom's color code: N = blue, C = turquoise, F = purple, and the yellow, lavender, and dark purple represent the peripheral N groups decorating the SubPc core.

The majority of the bowl-shaped polycyclic compounds studied so far present several significant drawbacks that have hampered their development in practical applications, including poor absorption in the solar spectrum, expensive and complex synthesis and purification processes, weak dipole moments, or low energy barriers for bowl-to-bowl racemization.^3,8,9^ In contrast, subphthalocyanines (SubPcs, Fig. 1), ring-contracted aza-porphyrinoids widely used as semiconductors and photosensitizers,^10^ are excellent candidates to overcome these material limitations. First, they are configurationally stable since the bowl-to-bowl inversion is blocked by the tetrahedral structure of the central boron atom. Second, their strong dipole moments are readily adjusted by chemical modifications. Third, they exhibit a strong – and also modulable – absorption in the visible range (500–600 nm), which opens their application in molecular photovoltaics, as either n- or p-type photo- and/or electro-active materials.^10,11^ Last but not least, they offer straightforward synthesis, purification, and derivatization. On this basis, several research groups have documented the synthesis and applications of SubPc-based columnar materials at different levels of order (*e.g.*, crystalline state, mesophases, or in solution).^12^ Despite this significant progress, a key aspect for the realistic technological development of these promising assemblies remains unclear: the structure/function relationship. In this regard, understanding how the substitution pattern of the building blocks directs the columnar assembly is arguably the most fundamental issue.

Herein, we study in detail the influence of two factors on the columnar organization of SubPcs: the number of peripheral substituents and chirality. To this end, we have synthesized three SubPcs (1-*Rac*, 1-*M* (and 1-*P*), and 2; Fig. 1) designed to form columnar polymers in solution. A comprehensive analysis of the stability and polymerization mechanism of the aggregates formed by these SubPcs has been conducted by means of experimental techniques, including variable-temperature (VT) and good-solvent (GS) experiments, as well as theoretical calculations involving molecular dynamics simulations with quantum mechanically derived force fields specifically tuned for SubPcs. Our work provides key insights into the self-assembly of bowl-shaped aromatics, a field which is still far from unleashing its full potential.

## Results and discussion

### Synthesis and general supramolecular behavior

**Scheme 1:**
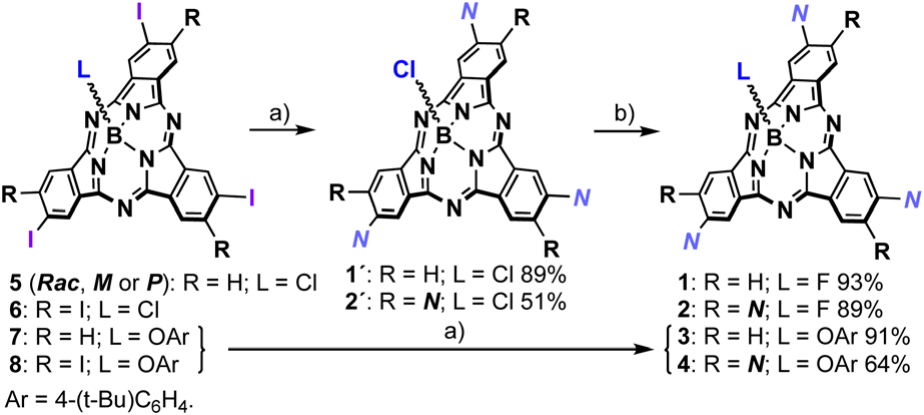
Synthesis of 1 (*Rac*, *M* or *P*) and 2. Reagents and conditions: (a) Pd_2_Cl_2_(PPh_3_)_2_, CuI, Et_3_N, N–H, THF, r.t., 12 h; (b) AgBF_4_, toluene, r.t., 12 h.

Inspired by hitherto reported supramolecular polymers involving π-components, we designed 1, in both the racemic (1-*Rac*) and enantiopure form (1-*M* and 1-*P*), and 2 as self-assembling units (Fig. 1). These derivatives consist of SubPcs β-substituted with either three or six fragments equipped with amide groups and long alkyl chains, which drive the polymerization by intermolecular hydrogen-bonding and van der Waals interactions, respectively. Unlike previous systems, 1 and 2 feature alkynes as bridges between the amides and the SubPc core, which were selected due to their conformational versatility and their ability to enable periphery-macrocycle electronic conjugation. On the other hand, a small fluorine atom was incorporated as the axial ligand to ensure efficient π–π stacking while reinforcing the aggregation by F⋯B dipole–dipole interactions. As illustrated in Scheme 1, 1-*Rac* and 2 were synthetized in good yields from 5 and 6, respectively, following a two-step sequence: (1) Sonogashira cross-coupling with N–H (Fig. 1); and (2) axial substitution using AgBF_4_ as chloride scavenger and fluoride source. 1-*M* were obtained similarly to 1-*Rac* but starting from 5 in its enantiopure form. Crucially, the enantiopurity of this sample remains unaltered throughout this process (see the ESI†), thereby highlighting the excellent configurational stability of SubPcs and anticipating the preparation of many other chiral materials *via* post-functionalization methods.

**Fig. 2:**
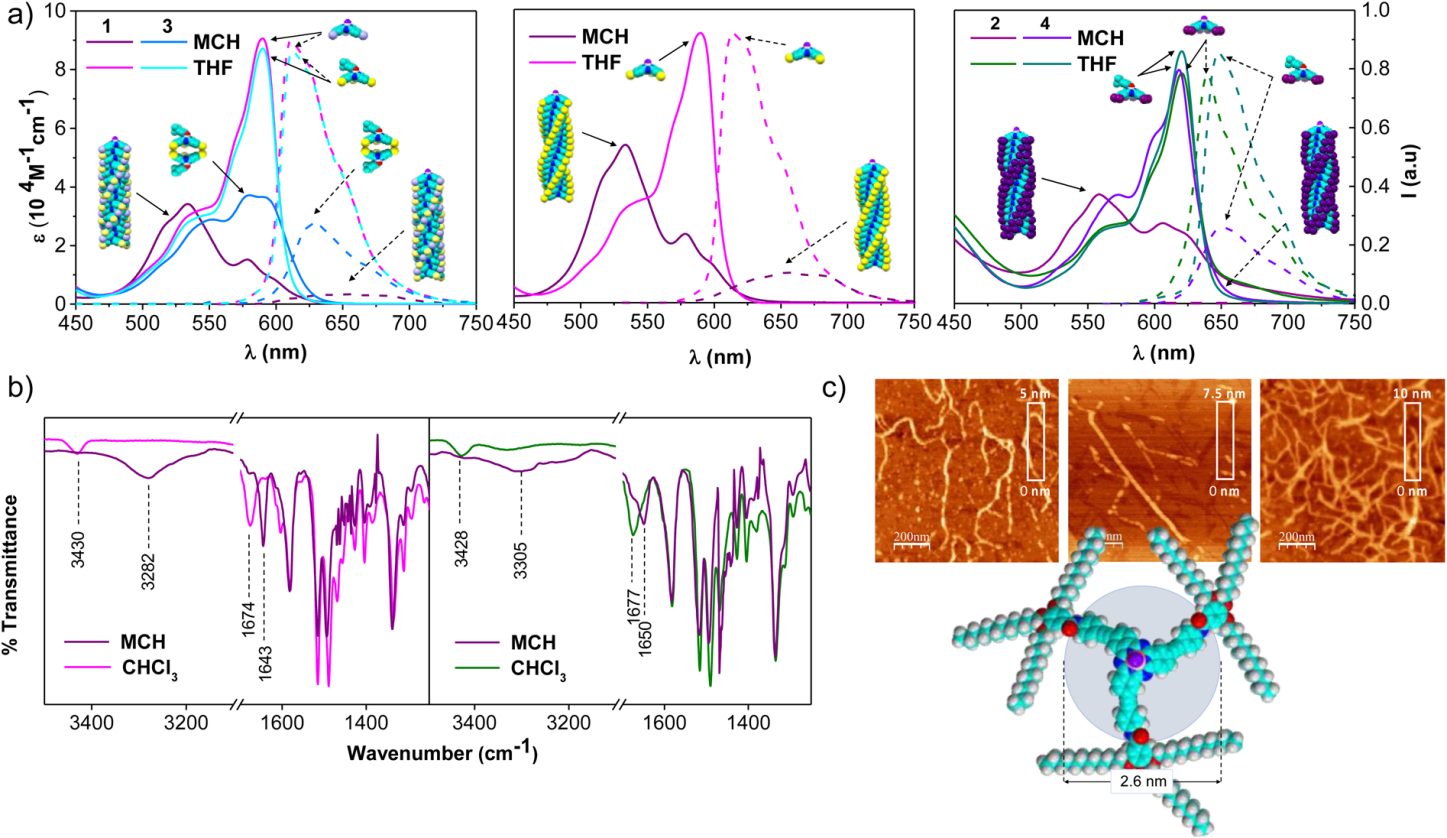
(a) Absorption (solid lines) and emission (dashed lines, *λ*_exc_ = 545 nm for tri-substituted SubPcs, and 580 nm for hexa-substituted) spectra of 1-*Rac* and 3 (left), 1-*M* (middle; 1-*P* shows similar behavior), and 2 and 4 (right) in THF and MCH ([SubPc] = 3.2 × 10^−5^ M). (b) FTIR spectra of 1 (left; racemic and enantiopure samples show similar spectra) and 2 (right) in CHCl_3_ and MCH ([SubPc] = 1.0 × 10^−3^ M). (c) From left-to-right, AFM images of the aggregates based on 1-*Rac*, 1-*M* and 2, and molecular model of 1.

The self-assembly ability of 1-*Rac*, 1-*M* (and 1-*P*, see ESI†), and 2 was initially assessed by recording the UV-vis spectra in tetrahydrofuran (THF), a good solvent that competes with the intermolecular H-bonds between the amide groups, and in methylcyclohexane (MCH), a non-polar solvent that induces the aggregation. As depicted in Fig. 2a, both 1-*Rac* and 1-*M* exhibit a narrow Q-band in THF peaking at 590 nm (*ε* = 91.520 M^−1^ cm^−1^) with progressive shoulders on the high-energy side, which is characteristic of non-aggregated SubPcs. The hexa-substituted derivative 2 exhibits a Q-band with a similar shape but red-shifted to 621 nm due to the conjugation induced by the additional alkyne groups. Regarding the emission properties, all three derivatives display strong emissions at 610 nm in the case of 1-*Rac* and 1-*M*, and at 639 nm in the case of 2. From these results, it can be concluded that 1-*Rac*, 1-*M*, and 2 exist as monomeric species in THF. In contrast, a substantially different spectroscopic scenario is observed in MCH. In this solvent, the Q-band of 1-*Rac*, 1-*M*, and 2 splits into two blue-shifted bands centered at 578 (*ε* = 22.400 M^−1^ cm^−1^) and 532 nm (*ε* = 53.600 M^−1^ cm^−1^) for 1-*Rac* and 1-*M*, and at 605 (*ε* = 26.800 M^−1^ cm^−1^) and 556 nm (*ε* = 36.815 M^−1^ cm^−1^) for 2. Furthermore, the fluorescence is significantly quenched. This spectral pattern is associated with the formation of SubPc-based columnar polymers, as indicated in previous studies.^12*f*^ To confirm this assumption, we prepared 3 and 4, which feature a bulky *tert*-butylphenoxy axial ligand instead of the fluorine atom to prevent head-to-tail interactions. In MCH, both the absorption and emission band of the three-substituted derivative 3 experiences a marked intensity decrease compared to THF, which is attributed to the formation of tail-to-tail dimers. By contrast, hexa-substituted 4 exhibits similar absorption and emission patterns in MCH and THF, suggesting that monomers are the predominant species in both solvents.

The involvement of H-bonding interactions in the supramolecular polymerization of 1-*Rac*, 1-*M*, and 2 was confirmed by registering the corresponding FTIR spectra in CHCl_3_ and MCH solutions (Fig. 2b). In CHCl_3_, the stretching N–H and C=O bands appear at 3430–3428 and 1677–1674 cm^−1^, respectively, which are characteristic wavenumber values of free amide (*i.e.*, dissociated monomers). In contrast, they are respectively located at lower wavenumber values (*ca.* 3305–3282 and 1650–1643 cm^−1^) in MCH, which are typical values of intermolecularly H-bonded amides and suggest the formation of columnar supramolecular polymers.^13^

The formation of columnar arrays was further demonstrated by atomic force microscopy (AFM) images on highly oriented pyrolytic graphite (HOPG) surfaces previously coated with MCH solutions of 1-*Rac*, 1-*M*, and 2. As shown in Fig. 2c and S6.1,† self-assembled 1-*Rac*, 1-*M*, and 2 revealed the formation of fibbers with heights (2.5–3.0 nm) that are consistent with the size of the SubPc core. Thus, we conclude that in MCH (and also in dodecane), 1-*Rac*, 1-*M*, and 2 form columnar supramolecular polymers based on a hydrogen-bond networking. It is important to note that, although both 1-*Rac* and 1-*M* show fibber-like organization, differences in morphology are noticeable. This finding suggests a different chemical composition, as demonstrated in VT and GS experiments (*vide infra*). NMR experiments at different temperatures in 1,2-tetrachloroethane (TCE) further confirm the ability of 1–2 to columnarly stack through overlapping of the π-conjugated cores (see the ESI† for further details).

## Supramolecular polymerization mechanism

**Fig. 3:**
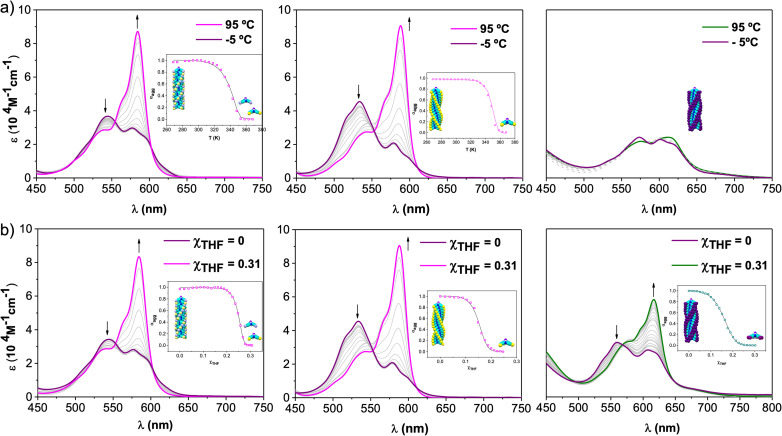
Absorption changes recorded for 1-*Rac* (left), 1-*M* (middle), and 2 (right) as a function of (a) temperature (scan rate = 0.5 K min^−1^, to ensure that the process takes place under thermodynamic control) and (b) solvent (THF/MCH) composition at 6.5 × 10^−6^ M. Arrows indicate spectral changes upon temperature increase or THF addition. Insets: evolution of the aggregation degree (*α*) *versus T* (a) or *χ*_THF_ (b) and the corresponding fit (solid line) to a nucleation–elongation model. This fit corresponds to a global fitting of three independent experiments at three different concentrations (see ESI† for further details).

To investigate the supramolecular polymerization mechanism governing the self-assembly of 1-*Rac*, 1-*M*, and 2, the absorption spectra of dilute solutions of these compounds in MCH ([SubPc] = 6.5 × 10^−6^ M) were recorded at different temperatures (Fig. 3a). Upon increasing the temperature from −5 to 95 °C, the spectra of 1-*Rac* and 1-*M* results in an absorption pattern comparable to that observed in THF (*i.e.*, solvated monomers). In contrast, the spectrum of 2 remains mainly unaltered suggesting the supramolecular polymer is not destroyed at 95 °C. This unexpected result points to a significant difference in the aggregate stability between tri- and hexa-substituted SubPcs. Indeed, the use of other solvents (dodecane, MCH/DCE or MCH/toluene mixtures) and higher temperatures led to similar results. Based on this finding, it can be concluded that the greater number of substituents capable of establishing intermolecular interactions (*i.e.*, hydrogen-bonding, π–π stacking, van der Waals, *etc.*) between monomers, the higher the stability of the entire assembly.

The corresponding cooling curves (*i.e.*, *α vs. T* plots; where *α* is the degree of polymerization and *T* the temperature) calculated from the extinction coefficient changes at *λ* = 576 nm for 1-*Rac* and 1-*M* are depicted in the insets of Fig. 3a. The role of chirality was quantitatively revealed by fitting these cooling curves to the cooperative nucleation–elongation model developed by Meijer, ten Eikelder, and co-workers (see Section S5.2 in the ESI†),^14^ which assumes that the polymerization takes place in two fundamental steps: (i) nucleation, where very short aggregates (dimers) are formed, and (ii) elongation, where such a nucleus elongates to form the final polymer. As can be seen in Fig. 3a, the data recorded for both 1-*Rac* and 1-*M* fitted well with a dimeric nucleus that then grows through successive SubPc stacking. This model allows us to obtain all the thermodynamic parameters that define the supramolecular polymerization, including nucleation enthalpy 
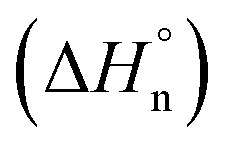
, elongation enthalpy (Δ*H*°) and entropy (Δ*S*°), nucleation (*K*_n_) and elongation (*K*_e_) binding constants, and the degree of cooperativity (*σ*). The values inferred for these thermodynamic parameters are collected in Table 1 (VT entries). Notably, 1-*Rac* and 1-*M* present noticeable differences. For instance, despite both polymerizations are highly cooperative, 1-*M* shows a *σ* value almost four times higher than that of 1-*Rac*. On the other hand, the entropy term of elongation in 1-*M* (−330 J mol^−1^ K^−1^) is more negative than that in 1-*Rac* (−130 J mol^−1^ K^−1^). Interestingly, although these Δ*S*° values are similar in other well-known π-systems (*e.g.*, in perylenes or tricarboxamides),^15^ they are more negative than those observed for the SubPc derivative in which the amide is directly linked to the aromatic core.^2*b*^ This indicates that SubPc 1, bearing alkynes that can rotate freely, is flexible to some extent. The enthalpic term is favored for 1-*M*, which displays a value (−149 kJ mol^−1^) almost twice that obtained for 1-*Rac* (−82 kJ mol^−1^). Interestingly, nucleation is much favored in 1-*M* by presenting a *K*_n_ = 8.1 × 10^3^ (2.0 × 10^3^ for 1-*Rac*), which is consistent with the lower cooperativity observed for 1-*M* compared with 1-*Rac*. These differences between the polymerization of 1-*Rac* and 1-*M* indicate that the supramolecular polymers formed should present a distinct chemical structure. Thus, the polymers based on 1-*M* are necessarily governed by head-to-tail homochiral interactions, whereas those composed of 1-*Rac* must involve an heterochiral binding. Further confirmation of this conclusion comes from AFM experiments, wherein a clear difference in the morphology and length of the fibers formed is noticeable. As confirmed by MD simulations (*vide infra*), heterochiral binding lead to more flexible polymers (entropically favored), whereas homochiral interactions gives rise to more robust H-bonding structures (enthalpically favored). A similar chiral social self-sorting has been previously observed with more rigid tri-substituted SubPcs, wherein the peripheral amide groups are closer to the aromatic core.^2*b*^

**Table 1:** Thermodynamic parameters calculated upon: polymerization by decreasing temperature in MCH (VT), and depolymerization in MCH : THF mixtures by increasing the volume fraction of THF (*χ*_THF_; GS)

		*K*_n_ a (M^−1^)	*K*_e_ b (M^−1^)	*T*_e_ c (K)	*σ* d	Δ*H*°^e^ (kJ mol^−1^)	Δ*S*°^f^ (J mol^−1^ K^−1^)	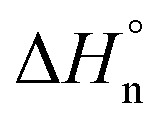 g (kJ mol^−1^)	*m* h (kJ mol^−1^)	Δ*G*°^i^ (kJ mol^−1^)
VT	1-*Rac*	2.0 × 10^3^	3.0 × 10^5^	348.1 ± 0.4	0.008	−82.0 ± 2.0	−130.0 ± 1.0	−13.9 ± 0.9		
VT	1-*M*	8.1 × 10^3^	2.5 × 10^5^	348.3 ± 0.5	0.032	−149.0 ± 6.9	−333.0 ± 2.0	−9.9 ± 1.1		
GS	1-*Rac*				0.002				123.0 ± 5.0	−66.2 ± 1.3
GS	1-*M*				0.036				98.7 ± 10.7	−43.8 ± 1.3
GS	2				1				86.2 ± 11.2	−48.1 ± 2.4

a Nucleation constant.

b Elongation constant.

c Elongation temperature.

d Degree of cooperativity.

e Elongation enthalpy.

f Elongation entropy.

g Nucleation enthalpy.

h *m* parameter.

i Gibbs free energy. VT and GS denote variable-temperature and good solvent experiments, respectively.

To further confirm the homochirality of 1-*M* polymers, we recorded their circular dichroism (CD) spectra upon increasing the temperature from −5 to 95 °C of a MCH solution ([SubPc] = 3.2 × 10^−5^ M, Fig. S5.2†). In line with the results obtained by UV-vis spectroscopy, a clear monomer-to-polymer transition was noticeable, where the CD spectra 95 and −5 °C are similar to those obtained in THF (dissolved monomer) and MCH (polymer) at room temperature, respectively. In THF, *M* and *P* enantiomers exhibit perfect mirror-image CD spectra with opposite Cotton effects (positive for the *M* enantiomer and negative for the *P* enantiomer) in the range of 300 to 600 nm. This CD response is consistent with that reported for other chiral SubPcs.^2*b*,12*a*,16^ In MCH, all signals significantly increase in intensity upon cooling, and the band peaking at 570 nm also experienced a blue-shift down to 530 nm. This spectral signature corresponds to the formation of columnar, homochiral polymers displaying helicity with a rotational sense (*M* or *P*) that coincides with the chirality of the enantiomer employed.

Further insights into the self-assembly of 1-*Rac*, 1-*M*, and 2 were obtained by analyzing the polymerization mechanism as a function of solvent composition. To conduct this study, we added increasing volumes of solutions of SubPcs in THF (*i.e.*, a good solvent) to solutions in MCH (*i.e.*, a bad solvent), keeping the total concentration constant at 6.5 × 10^−6^ M. A similar spectral change to that observed in VT experiments is observed for 1-*Rac* and 1-*M* upon the addition of increasing amounts of THF (Fig. 3b). Importantly, in the case of 2, the absorption pattern of the monomer is also recovered when adding THF and the dissolved molecular state is achieved. This experiment allowed us for obtaining denaturation curves of 1-*Rac*, 1-*M*, and 2, which were later analyzed by means of the extended nucleation-elongation model developed by de Greef, Meijer, and co-workers (see Section S5.3 of the ESI†).^17^ Thus, by plotting the variation of *α versus χ*_THF_, we obtained Δ*G*_0_, *m*, and *σ*, with Δ*G*_0_ representing the Gibbs free energy gain upon monomer addition in the pure solvent (MCH) and *m* the ability of the good solvent to associate with the monomer. Fortunately, clear polymer-to-monomer transitions upon addition of THF aliquots were observed for both 1-*Rac*, 1-*M*, and 2. The corresponding THF/MCH spectral evolution, denaturation curves, and fittings, along with the thermodynamic parameters obtained, are presented in Fig. 3b and Table 1 (GS entries), respectively. In line with the trends observed in VT experiments, 1-*Rac* and 1-*M* polymerize *via* cooperative mechanism, with the *σ* value being higher for the pure enantiomers. In sharp contrast, 2 displays a *σ* value of 1, which is diagnostic of an isodesmic mechanism. This work represents, therefore, a paradigmatic example of how the number of peripheral substituents can modulate the supramolecular polymerization mechanism of bowl-shaped aromatics. Regarding Δ*G*_0_, 1-*Rac* exhibited the most spontaneous elongation, followed by 2 and 1-*M*, which had similar values. Interestingly, the ability of THF to disassemble the polymer based on 1-*Rac* is higher than that of 2 and 1-*M*. It should be noted that the higher amount of THF required to denature polymers made of 1-*M* compared to 1-*Rac* points out to a different composition (*i.e.*, homochiral and heterochiral, respectively), which is in line with VT experiments.

### Theoretical analysis of self-assembly

Molecular dynamics (MD) simulations^18^ were utilized to properly describe the supramolecular structures formed by 1-*Rac*, 1-*M*, and 2 accounting, simultaneously, for conformational disorder (see the ESI† for full details of the computational protocol). The degree of detail necessary to this aim cannot be achieved with standard force fields, which are not tuned for SubPcs. No specific force field for SubPcs was indeed found in the literature, and a quantum mechanically derived force field (QMDFF) based on DFT calculations performed at the B3LYP/cc-PVDZ level of theory was developed with the Joyce code.^19^ The very accurate results obtained for the parameterization allows to consider the generated QMDFF on equal terms than the DFT level used as reference (see the ESI† for details).

Two molecular models were employed in the MD simulations: a head-to-tail dimer (1_2_) as representative of the polymerization nucleus, and a head-to-tail octamer (1_8_) as model of the supramolecular polymer. For the latter, special attention was paid to the central dimer of the octamer (1_8(2)_) because terminal effects are minimized for it, thus providing a closer description of the supramolecular polymer. For the racemic mixture, it was assumed that the two enantiomers (1-*M* and 1-*P*) fully alternate along a heterochiral column.

**Fig. 4:**
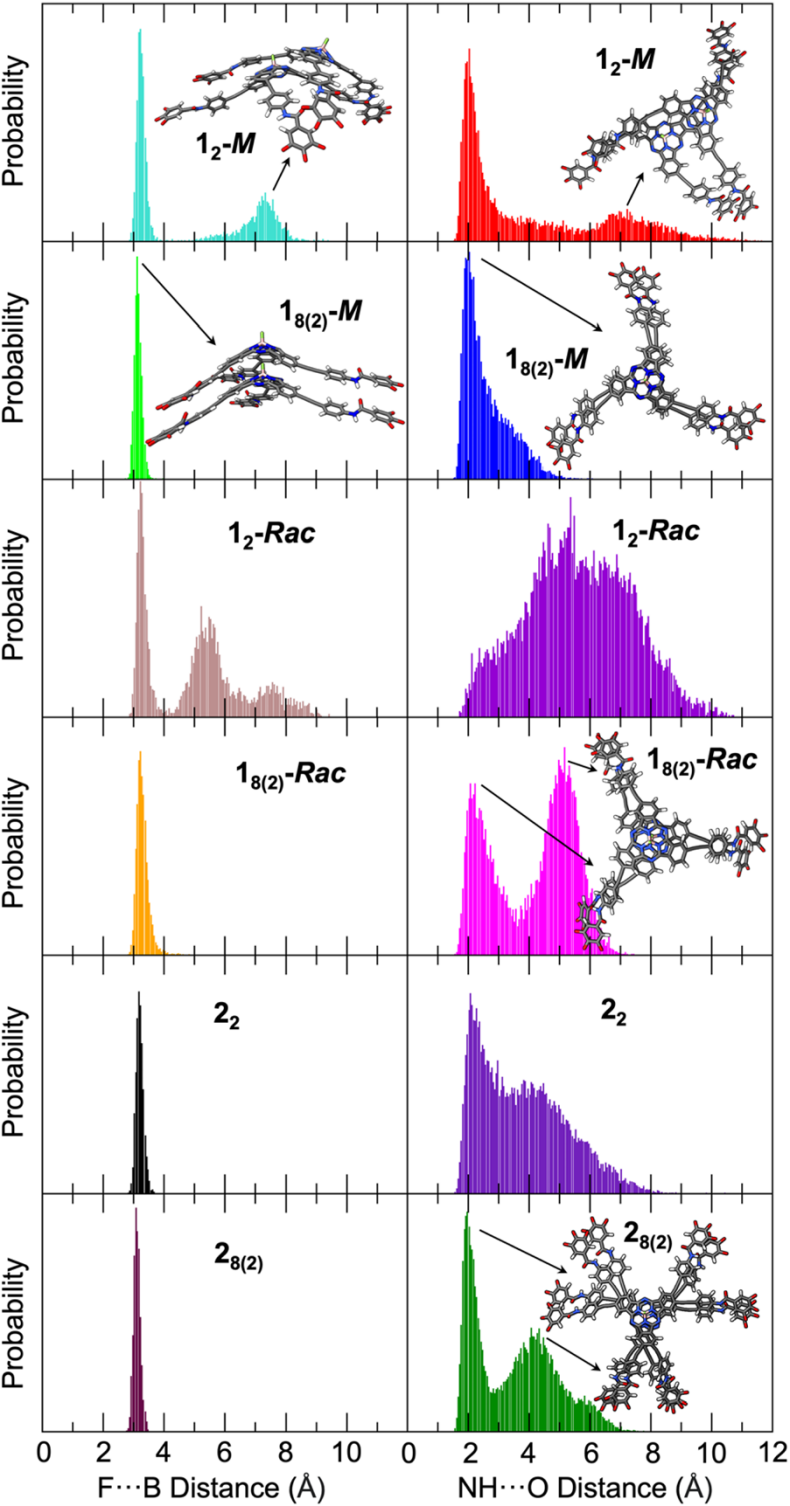
Distribution of the intermolecular F⋯B (left) and amide NH⋯O (right) distances of (from top to bottom) 1_2_-*M*, 1_8(2)_-*M*, 1_2_-*Rac*, 1_8(2)_-*Rac*, 2_2_ and 2_8(2)_ along the MD simulations. Data from 4001 structures, all the three amides were considered for NH⋯O distances. Representative examples of the structures corresponding to the distribution maxima are shown. In the bottom right panel, one of the three amide NH⋯O bonds is formed (top substituent) while on the other two the π-stacking dominates, enlarging the H bond distance.

The most relevant structural parameters to follow the evolution of the columnar aggregate are the intermolecular F⋯B and amide NH⋯O distances between adjacent SubPcs. Fig. 4 shows the distribution of these distances along the MD simulation for 1_2_-*M* and 1_8(2)_-*M*. Notably, a bimodal distribution is observed for both distances in 1_2_-*M*, which includes a global maximum at short distances (3.2 and 2.0 Å for F⋯B and H-bonds, respectively), and a second local broader maximum at longer distances (7.3 and 6.8 Å, respectively). The former is characteristic of a well-formed head-to-tail dimer involving the formation of intermolecular H-bonds between the three amide groups. The latter corresponds to partially dissociated structures where the two SubPcs interact directly through the π surfaces without the F⋯B interaction, a first step prior to dissociation (see Fig. S7.5 in the ESI† for structural models). In contrast, the columnar structure of 1_8(2)_-*M* is preserved during the whole MD simulation showing a very narrow distribution for F⋯B distances with values of 3.15 ± 0.12 Å. Concerning H-bonds, the distribution maximum is at 2.0 Å, although they can transiently be broken and reestablished as inferred by the non-negligible population in the 3–5 Å range. These data suggest that the stability of the nuclei for polymerization (1_2_-*M* model) might be compromised when the number of monomers is small, but it steadily becomes more robust upon the size of the aggregate grows up (1_8(2)_-*M* model). This theoretical result supports the much smaller value inferred experimentally for *K*_n_ in comparison with *K*_e_ (Table 1).

When comparing 1_2_-*Rac* and 1_8(2)_-*Rac*, the F⋯B distances show a similar trend: whereas the columnar aggregate is well-preserved for 1_8(2)_-*Rac*, partial dissociation occurs for 1_2_-*Rac*. However, the differences found for the amide H-bonds are notable. For 1_2_-*Rac*, the distribution is quite irregular and the H-bond distances range from 2.0 up to 10.0 Å, meaning that the H-bonds can be formed but are mainly broken during most of the trajectory. This results in a smaller stability of the dimer with respect to the homochiral case, which is in good agreement with the smaller *K*_n_ value deduced for 1-*Rac* compared with 1-*M* (Table 1). On the other hand, a clear bimodal distribution is observed for 1_8(2)_-*Rac*, with two maxima around 2.1 and 5.2 Å. Whereas the first maximum is typical of well-formed H-bonds, the second maximum corresponds to structures in which the H-bonds have been replaced by the π-stacking of the external phenyl rings, driven by the rotation of the amide group and being in competition with the H-bonds (see the structural model of Fig. 4 and in more detail Fig. S7.6†). However, this process is apparently too fast to be appreciated in the FTIR spectra of 1-*Rac* and 1-*M*, which are identical. The origin of the differentiated behavior of 1-*Rac* and 1-*M* can be understood from a structural point of view. The enantiomerically pure 1-*M* allows a maximum overlap of the SubPc aromatic system while keeping the most favorable arrangement for amide H-bonding. In contrast, for 1-*Rac*, the optimal H-bonded structure of the heterochiral columnar aggregate impedes the π-stacking of the SubPcs, and *vice versa*, thus resulting in a competition between both interactions that lead to a more dynamical structure for 1-*Rac*. In other words, polymers made of 1-*M* are enthalpically favored, whereas those made of 1-*Rac* are entropically favored. Thus, the entropic penalty of homochiral interaction must make the heterochiral polymerization preferred in the racemic mixture, rather than homochiral. This conclusion is in excellent agreement with the thermodynamic parameters experimentally observed. See Fig. S7.7† for the optimized structures of homochiral 1_8(2)_-*M* and heterochiral 1_8(2)_-*Rac*. Fig. S7.8 and S7.9† show the more regular and compact columnar self-assembly achieved for 1-*M* compared to 1-*Rac*.

Fig. 4 also shows the theoretical results obtained for the hexa-substituted compound 2 from MD simulations. As a whole, MD calculations predict much more stable aggregated structures for 2 than for 1 due to the larger number of interactions, which is in good correlation with the experimental results. For instance, the F⋯B distance has a single maximum at 3.17 Å for the 2_2_ dimer, which shortens to 3.08 Å for the more rigid supramolecular octamer. Thus, no partial dissociation is observed in contrast to that found for 1_2_-*Rac* and 1_2_-*M*. The amide H-bond distances, although showing a broad distribution in the 1.5–7.5 Å range, display narrow maxima at 2.07 and 1.97 Å for 2_2_ and 2_8(2)_, respectively. In fact, the distribution is much more displaced towards the global maximum than in 1-*Rac* and 1-*M*, suggesting a stronger H-bond network. The second maximum at 4.2 Å is associated to the amide rotation and shows some remaining conformational freedom. Therefore, according to these results it is reasonable to assume that *K*_n_ is much larger for 2 than for 1-*Rac* or 1-*M*, which indeed would give rise to a higher *σ* value in good agreement with the experimental evidence in GS experiments (Table 1). Fig. S7.10† shows the crowded packing obtained for 2 upon columnar aggregation.

Finally, the capability of 1 and 2 to form tail-to-tail dimers, identified in the literature for similar trisubstituted SubPcs, was also analyzed. As shown in Fig. S7.11,† the crowded structure of the hexa-substituted derivative 2 causes a high steric hindrance between the six peripheral groups, which results in a very weak interaction between the SubPcs with a B⋯B distance of 16.01 Å. In contrast, for 1-*M* and 1-*Rac* the three substituents of each SubPc can accommodate between them and gives rise to a close interacting tail-to-tail dimer with a B⋯B distance of 7.03 Å. These results explain the fact that for the formation of tail-to-tail dimers is observed in MCH for the three-substituted derivative 3 but not for the hexa-substituted derivative 4 (Fig. 2a).

## Conclusions

In summary, the role of chirality and peripheral substitution in the columnar organization of SubPcs has been unveiled. The synergy between different spectroscopic studies have revealed that 1-*Rac*, 1-*M*, and 2 self-assemble in non-polar solvents by hydrogen-bonding leading to the formation of columnar polymers which, depending on the number of peripheral amide substituents born by the molecular building block, differs in stability and formation mechanism. The polymerization process has been analyzed as a function on temperature for 1-*Rac* and 1-*M*, and solvent composition, for 1-*Rac*, 1-*M* and 2. In variable temperature conditions, both 1-*Rac* and 1-*M* polymerize *via* a cooperative mechanism, although in the latter case the *σ* value is four times higher. 1-*Rac* forms polymers with an alternate stacking of enantiomers (*i.e.*, heterochiral), whereas 1-*M* self-assembles into homochiral arrays. In MCH/THF mixtures at r.t., the polymers based on 1-*Rac* and 1-*M* are formed following a cooperative nucleation-elongation process, whereas 2, remarkably, follows an isodesmic mechanism. Molecular dynamics simulations have revealed that the differences between 1-*Rac*, 1-*M*, and 2 rely on structural effects. In 1-*M* (*i.e.*, homochiral columnar polymers), the SubPc π-skeleton can overlap without preventing the formation of hydrogen-bonds, leading to a more enthalpically favored polymerization. By contrast, in the case of 1-*Rac* (*i.e.*, heterochiral columnar polymers), this overlapping induces a non-optimal orientation of the amides for hydrogen-bonding, which results in a less robust SubPc–SubPc linking and a more entropically favored packing. This effect makes the nucleus formation (*K*_n_) for 1-*Rac* not so favorable as for 1-*M*, which explains the higher cooperativity in the self-assembly of 1-*Rac*. On the other hand, the nucleus formation is highly exergonic in the case of 2 due to the additional intermolecular interactions, which is in line with its isodesmic behavior.

In conclusion, this work provides valuable insights into the columnar assembly of SubPcs that we expect will contribute to the still-nascent exploitation of bowl-shaped aromatics in materials science. The properties and functions of these assemblies are currently being studied in our laboratories.

## Data availability

The data supporting the findings of this study are available within the article and its ESI.† The details about MD simulations are available in additional ESI files.†

## Author contributions

J. L., E. O. and T. T. designed the research. J. L. and E. L.-S. performed the synthesis and self-assembly studies. M. J. M. performed self-assembly studies. D. A. performed the theoretical studies. All authors contributed to the writing and editing of the paper. Overall, J. L., E. L.-S. and D. A. contributed equally to this publication.

## Conflicts of interest

There are no conflicts to declare.

## Supplementary Material

SC-015-D4SC03976A-s001

SC-015-D4SC03976A-s002

## References

[cit1] Meijer E. W., Schenning A. P. H. J. (2002). Nature.

[cit2] Wang L., Huang D., Lam L., Cheng Z. (2017). Liq. Cryst. Today.

[cit3] Martin J., Slavchov R., Yapp E., Akroyd J., Mosbach S., Kraft M. (2017). J. Phys. Chem. C.

[cit4] Miyajima D., Tashiro K., Araoka F., Takezoe H., Kim J., Kato K., Takata M., Aida T. (2009). J. Am. Chem. Soc..

[cit5] Zhang C., Nakano K., Nakamura M., Araoka F., Tajima K., Miyajima D. (2020). J. Am. Chem. Soc..

[cit6] Szumna A. (2010). Chem. Soc. Rev..

[cit7] Brandt J. R., Salerno F., Fuchter M. J. (2017). Nat. Rev. Chem..

[cit8] Saito M., Shinokubo H., Sakurai H. (2018). Mater. Chem. Front..

[cit9] Higashibayashi S. , Chapter 4-Control of Inversion Kinetics of Bowl-Shaped Aromatic Compounds, Academic Press, 2019, pp. 65–96

[cit10] Claessens C. G., González-Rodríguez D., Rodríguez-Morgade M. S., Medina A., Torres T. (2014). Chem. Rev..

[cit11] Labella J., Shoyama K., Guzmán D., Schembri T., Stolte M., Torres T., Würthner F. (2023). ACS Mater. Lett..

[cit12] Labella J., Lavarda G., Hernández-López L., Aguilar-Galindo F., Díaz-Tendero S., Lobo-Checa J., Torres T. (2022). J. Am. Chem. Soc..

[cit13] Wehner M., Röhr M. I. S., Böhler M., Stepanenko V., Wagner W., Würthner F. (2019). J. Am. Chem. Soc..

[cit14] Markvoort A. J., ten Eikelder H. M. M., Hilbers P. A. J., de Greef T. F. A., Meijer E. W. (2011). Nat. Commun..

[cit15] Martínez M. A., Doncel-Giménez A., Cerdá J., Calbo J., Rodríguez R., Aragó J., Crassous J., Ortí E., Sánchez L. (2021). J. Am. Chem. Soc..

[cit16] Shimizu S., Miura A., Khene S., Nyokong T., Kobayashi N. (2011). J. Am. Chem. Soc..

[cit17] Korevaar P. A., Schaefer C., de Greef T. F. A., Meijer E. W. (2012). J. Am. Chem. Soc..

[cit18] Abraham M. J., Murtola T., Schulz R., Páll S., Smith J. C., Hess B., Lindahl E. (2015). SoftwareX.

[cit19] Cacelli I., Prampolini G. (2007). J. Chem. Theory Comput..

