# Supplementary material for: The key role of chirality and peripheral substitution in the columnar organization of bowl-shaped subphthalocyanines
Source: Chem Sci. 2024 Jul 29;15(34):13760–7. doi: 10.1039/d4sc03976a (PMC11352688; doi:10.1039/d4sc03976a)
Supplement: SC-015-D4SC03976A-s001 [file SC-015-D4SC03976A-s001.pdf]

Supplementary Information (SI) for Chemical Science.
This journal is © The Royal Society of Chemistry 2024

Supplementary Information

The Key Role of Chirality and Peripheral Substitution in the Columnar Organization of Bowl-Shaped Subphthalocyanines

Jorge Labella,* † [a] Elisa López-Serrano, † [a] Daniel Aranda, † [b,c] María J. Mayoral,[d] Enrique Ortí,* [b] and Tomás Torres* [a,e,f]

[a] Department of Organic Chemistry, Universidad Autónoma de Madrid, Campus de Cantoblanco. C/ Francisco Tomás y Valiente 7, 28049 Madrid (Spain)

[b] Instituto de Ciencia Molecular (ICMol), Universidad de Valencia 46980 Paterna, Spain

[c] Andalucía Tech, Facultad de Ciencias, Departamento de Química Física, Universidad de Málaga, 29071-Málaga, Spain.

[d] Inorganic Chemistry Department, Universidad Complutense de Madrid, 28040 Madrid, Spain

[e] Institute for Advanced Research in Chemical Sciences (IAdChem), Universidad Autónoma de Madrid, Madrid (Spain).

[f] IMDEA – Nanociencia C/ Faraday 9, Campus de Cantoblanco, 28049 Madrid (Spain)

†These three authors contributed equally to this work

Table of contents

1. Materials and methods 2
2. Synthetic Procedures and Compound Data 3
3. NMR spectra 8
4. Mass spectra 12
5. Additional aggregation studies 14
   5.1. NMR Studies 14
   5.2. Nucleation-elongation model for cooperative supramolecular polymerization from variable temperature experiments 15
   5.3. Nucleation-elongation model for cooperative supramolecular polymerizations from good solvent experiments 15
   5.4. Aggregation studies of 1-*P* 16
   5.5. Fits to a nucleation−elongation model at different concentrations 16
   5.5. CD aggregation studies 17
6. AFM Measurements 18
7. Computational studies 19
   7.1. Force Field Parametrization 19
   7.2. Computational Details 25
   7.3. Additional results 26

1. Materials and methods

The monitoring of the reactions was carried out by thin layer chromatography (TLC), employing aluminum sheets coated with silica gel type 60 F254 (0.2 mm thick, Merck). The analysis of the TLCs was performed with an UV lamp of 254 and 365 nm. Purification and separation of the synthesized products were performed by normal-phase column chromatography, using silica-gel (230–400 mesh, 0.040–0.063 mm, Merck). Eluents along with the relative ratio in the case of solvent mixtures are indicated for each particular case. Nuclear magnetic resonance spectra (^{1}H-, ^{13}C-, ^{11}B-, and ^{19}F-NMR) were recorded on Bruker AV-300, Bruker AV III HD 400 MHz, or Bruker DRX-500 spectrometers. The deuterated solvent employed in each case is indicated within brackets, and its residual peak was used to calibrate the spectra using literature reference δ ppm values.[1] All the NMR spectra were recorded at room temperature. High-resolution mass spectra (HRMS) were obtained at the Interdepartmental Investigation Service of UAM or IOC mass spectrometry lab of University of Würzburg, employing matrix-assisted laser desorption/ionization time-of-flight (MALDI-TOF) using a Bruker-Ultraflex-III spectrometer, with a Nd:YAG laser operating at 355 nm or an ultrafleXtreme spectrometer, or an ESI TOF using a Bruker Daltonics microTOF focus instrument. The matrixes and internal references employed are indicated for each spectrum. Infrared spectra were recorded in solid state on a Bruker Vector 22 spectrophotometer. Ultraviolet and visible (UV-Vis) spectra were recorded using solvents of spectroscopic grade in the Organic Chemistry Department of UAM employing JASCO-V660 and PerkinElmer Lambda 2 dual beam absorption spectrophotometers. The logarithm of the molar extinction coefficient (ε) is indicated in brackets for each maximum. Likewise, fluorescence measurements were carried out with a JASCO-V8600 spectrofluorometer and a Horiba Jobin Yvon FluoroMax-3 emission spectrometer. NIR emission spectra were recorded on a Horiba Jobin Yvon FluoroLog3 spectrometer using a 450 W xenon lamp and a Symphony InGaAs array detector in combination with an iHR320 imaging spectrometer. CD spectra were recorded with a JASCO V-815 equipment. Resolution of racemic I_3-SubPc-Cl was carried out by chiral high-performance liquid chromatography (HPLC) using an Agilent 1200 equipment with a semi-preparative Daicel Chiralpak IC column (10 mm ø x 20 mm). Chemicals were purchased from commercial suppliers and used without further purification. FTIR spectra in solution were recorded on a Jasco FT-IR4600 spectrometer using a CaF_2 cell with a path length of 0.1 nm. Dry solvents were purchased from commercial suppliers in anhydrous grade or thoroughly dried before use employing standard methods. Solid, hygroscopic reagents were dried in a vacuum oven before use. Atomic force microscopy (AFM) images were recorded with a Ntegra Prima (NT-MDT) instrument at the Instituto Madrileño de Estudios Avanzados de Nanociencia (IMDEA), in tapping mode.

2. Synthetic Procedures and Compound Data

The synthesis and characterization of 3,4,5-tris(dodecyloxy)-*N*-(4-ethynylphenyl)benzamide (*N*–H)[2] and subphthalocyanines **5**,[3] **7**,[4] **8**,[5] and **6**[6] have been previously reported.

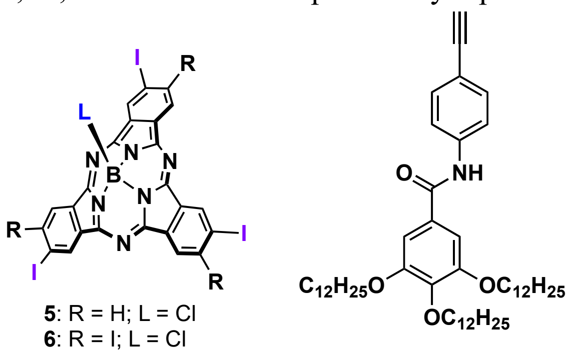

5: R = H; L = Cl
6: R = I; L = Cl
7: R = H; L = OPh
8: R = I; L = OPh

N–H

<u>Synthesis and characterization of SubPcs **1-4**</u>

SubPcs **1-*Rac***, ***M*** and ***P***

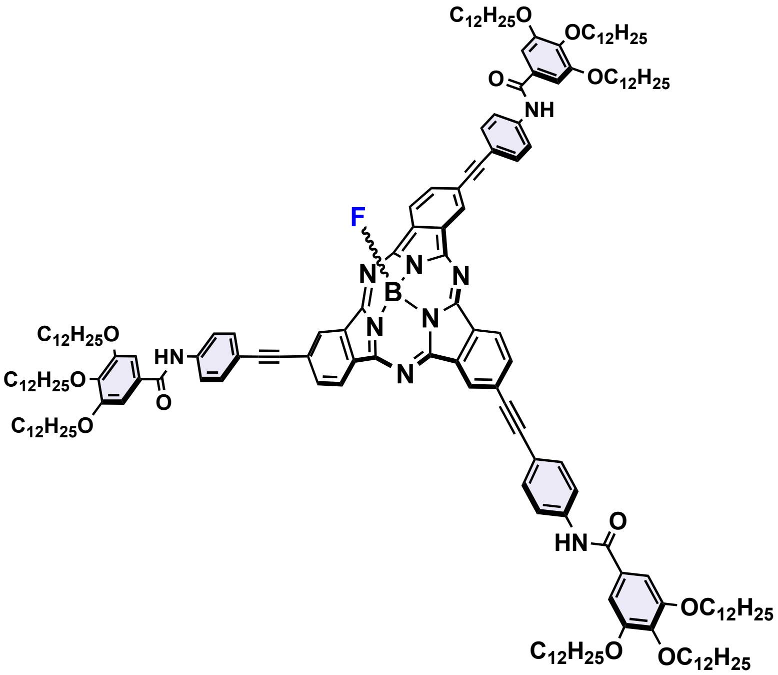

In a 25 mL Schlenk flask, equipped with a magnetic stirrer, **5** (*Rac*, *M* or *P*; 40 mg, 0.051 mmol), $PdCl_2(PPh_3)_2$ (7.0 mg, 0.010 mmol), CuI (1.7 mg, 0.010 mmol), and *N*–H (140 mg, 0.15 mmol) were placed under argon atmosphere. Then, 3 mL of a mixture of THF/NEt_3 5:1, which was deoxygenated via three freeze-pump-thaw cycles, was added and the resulting mixture was stirred at room temperature overnight. After that, the crude was dissolved in DCM and passed through a short celite plug. The solvent was removed by vacuum distillation and the resulting dark solid was purified by column chromatography on silica gel using DCM/MeOH 100:1 as eluent. Upon recrystallization from methanol, **1´**(See Scheme 1 in the main text) was obtained as a pink solid (123 mg, 0.045 mmol). In a 10 mL Schlenk flask, equipped with a magnetic stirrer, **1´** (50 mg, 0.018 mmol) and silver tetrafluoroborate (19 mg, 0.098 mmol) were placed under argon atmosphere. Then, 2 mL of dry toluene was added, and the mixture was stirred at r.t. for 12 h. After this, the solvent was then eliminated under reduced pressure and the residue purified by column chromatography on silica gel

(eluent: DCM/MeOH 100:1) and then by size exclusion chromatography using Bio-beads and $CHCl_3$ as eluent. Upon recrystallization from methanol, **1** was obtained as a purple solid (46 mg, 0.017 mmol). Yield: 93 %. **^{1}H-NMR** (300 MHz, THF-d_8): δ (ppm) = 9.40 (s, 3H, NH), 8.99 (s, 3H), 8.82 (d, $^3J_{H\text{-}H}$ 8.225 Hz, 3H), 8.05 (dd, $^4J_{H\text{-}H}$ = 1.47 Hz, 3H), 7.85 (d, $^3J_{H\text{-}H}$ = 8.76, 6H), 7.59 (d, $^3J_{H\text{-}H}$ = 8.61, 6H), 7.24 (s, 6H), 4.06 (t, 12H), 4.00 (t, 6H), 1.83 (m, 18H), 1.59-1.48 (m, 18H), 1.46-1.2 (m, 144 H), 0.89 (t, 27 H); **^{13}C-NMR** (75.5 MHz, THF-d_8): δ (ppm) = 165.00, 153.00, 151.67, 141.67, 140.57, 132.07, 130.08, 125.46, 119.57, 117.74, 106.60, 95.56, 88.49, 72.76, 69.09, 66.95, 66.77, 66.60, 66.42, 66.24, 66.07, 31.93, 31.91, 30.42, 29.80, 29.77, 29.71, 29.70, 29.67, 29.61, 29.53, 29.45, 29.39, 29.36, 26.17, 24.83, 24.66, 24.50, 24.43, 24.34, 24.27, 24.18, 24.02, 22.59, 13.46; **^{11}B-NMR** (160.5 MHz, THF-d_8): δ (ppm) = –13.66 (d, J = 28.89 Hz, 1B; B—F); **^{19}F-NMR** (470 MHz, THF-d_8: δ (ppm) = –157.61 (q, J = 32.9 Hz ,1F; B—F); **MS** (MALDI-TOF, DCTB): m/z = 2730.0; [M]+. HRLSI-MS (DCTB + PPGNa 2000 + PPGNa 2700): m/z Calculated for [$C_{177}H_{255}BFN_9O_{12}$]: 2729.9738; Found: 2729.9764; **UV/vis** (THF): λ_{max} (nm) (log ε (dm^3 mol^{-1} cm^{-1})) = 587 (8.6), 539 (3.0), 375 (5.2).

HPLC analysis of 1-*M* and *P*

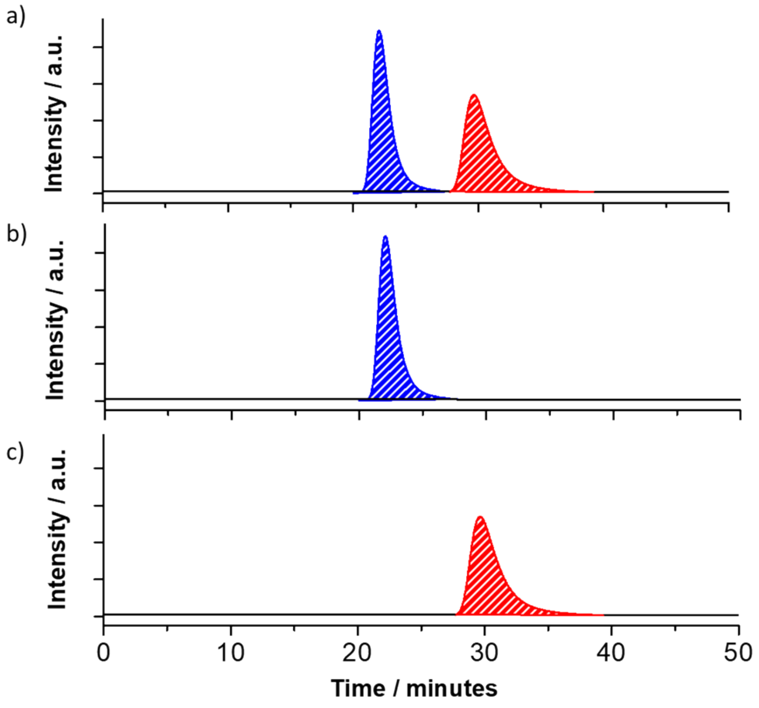

Figure S2.1. a) HPLC chromatogram of **1-*Rac*** with peaks corresponding to the *M* (blue trace) and *P* (red trace) enantiomers. The percentage area underneath the first and second peak is 50.0% in both cases. b-c) HPLC chromatogram of the corresponding pure enantiomers. Eluting solvents = toluene/hexane/AcOEt 60:30:10; flow rate = 1 mL min^{-1}; temperature = 20 ºC, detection wavelength = 560 nm.

SubPc **2**

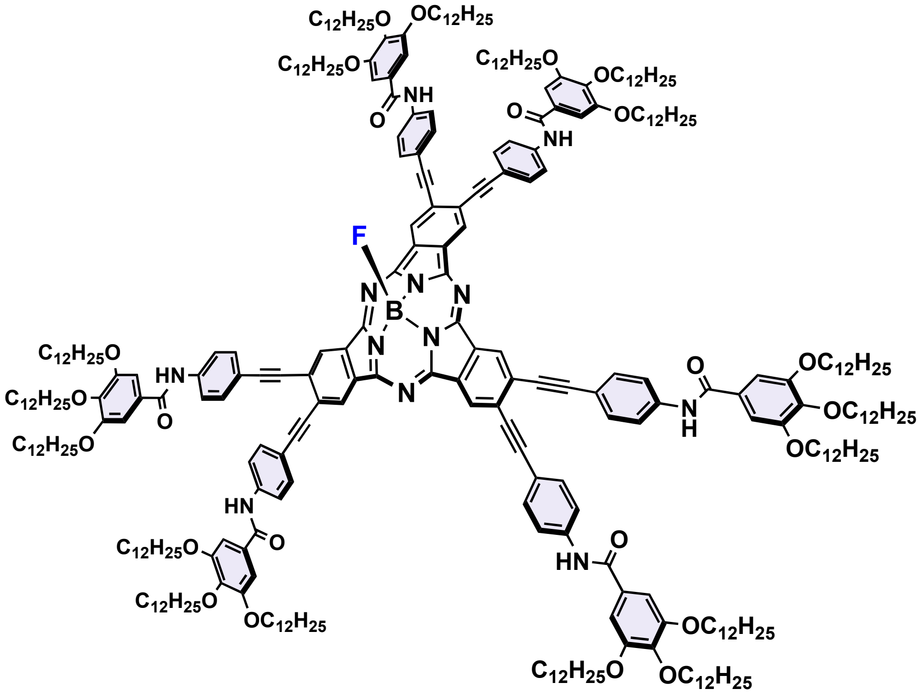

In a 25 mL Schlenk flask, equipped with a magnetic stirrer , **6** (30 mg, 0.026 mmol), $PdCl_2(PPh_3)_2$ (7.2 mg , 0.0010 mmol), CuI (3.9 mg, 0.0021 mmol), and *N*–H (143 mg, 0.19 mmol) were placed under argon atmosphere. Then, 4.5 mL of a mixture of THF/NEt_3 5:1, which was deoxygenated via three freeze-pump-thaw cycles, was added and the resulting mixture was stirred first at 50 ºC for 30 min, and then at room temperature overnight. After that, the crude was dissolved in DCM and passed through a short celite plug. The solvent was removed by vacuum distillation and the resulting dark green solid was purified by column chromatography on silica gel using DCM/MeOH 100:1. Thus, **2´** was obtained as a green viscous solid (67 mg, 0.013 mmol). In a 10 mL Schlenk flask, equipped with a magnetic stirrer, **2´** (66 mg, 0.013 mmol) and silver tetrafluoroborate (25 mg, 0.13 mmol) were placed under argon atmosphere. Then, 2.5 mL of dry toluene was added and the mixture was stirred at r.t. for 12 h. After this, the solvent was then eliminated under reduced pressure and the residue purified by column chromatography on silica gel (eluent: DCM/MeOH 40:1) and by size exclusion chromatography using Bio-beads and $CHCl_3$ as eluent. Upon recrystallization from methanol, **2** was obtained as a green solid (60 mg, 0.012 mmol). Yield: 89 %; **^{1}H-NMR** (300 MHz, THF d_8): δ (ppm) = 9.44 (s, 6H, NH), 9.00 (s, 6H), 7.87 (d, $^3J_{H-H}$ = 8.64 Hz, 12H), 7.67 (d, $^3J_{H-H}$ = 8.55 Hz, 12H), 7.25 (s, 12H), 4.05 (t, 24H), 4.00 (t, 12H), 1.87–1.80 (m, 82H), 1.61–1.48 (m, 290H), 0.90 (t, 54H); **^{13}C-NMR** (75.5 MHz, THF d_8): Due to the high molecular weight and the limited solubility of this molecule, the acquisition of this spectrum was not possible; **^{11}B-NMR** (160.5 MHz, THF d_8): δ (ppm) = –13.45 (d, J = 28.89 Hz, 1B; B─F); **^{19}F-NMR** (470 MHz, THF d_8: δ (ppm) = –156.80 (q, J = 32.9 Hz, 1F; B─F); **MS** (MALDI-TOF, DCTB): m/z = 5046.7 [M]+; **HRLSI-MS** (DCTB + PPGNa 5000): m/z Calculated for [$C_{330}H_{498}BFN_{12}O_{24}$]: 5046.8301; Found: 5046.8256; **UV/vis** (THF): λ_{max} (nm) (log ε (dm^3 mol^{-1} cm^{-1})) = 620 (9.5), 566 (3.4), 321 (4.3).

SubPc **3**

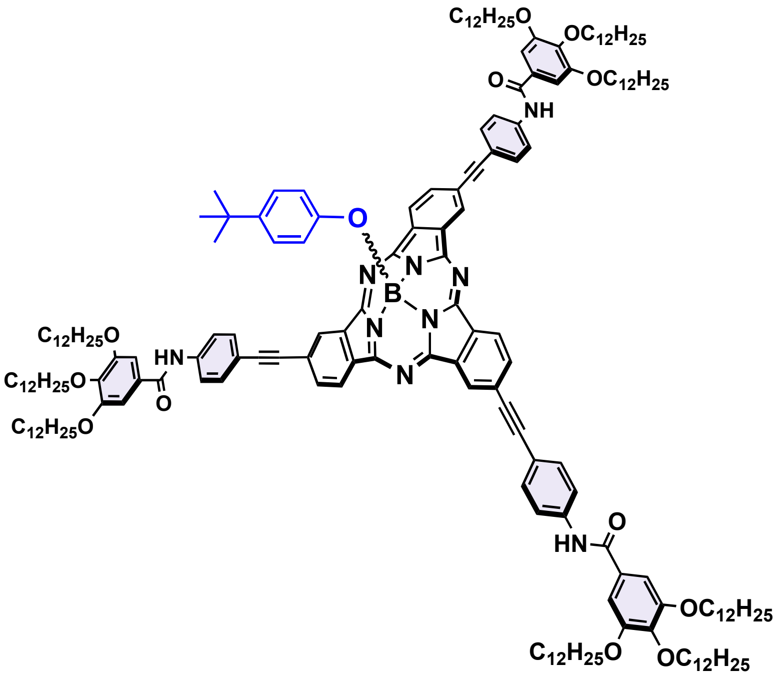

In a 25 mL Schlenk flask, equipped with a magnetic stirrer, **7** (20 mg, 0.022 mmol), $PdCl_2(PPh_3)_2$ (3.0 mg, 0.0044 mmol), CuI (1.5 mg, 0.0078 mmol) and *N*–H (61 mg, 0.078 mmol) were placed under argon atmosphere. Then, 1.5 mL of a mixture of THF/NEt_3 5:1, which was deoxygenated via three freeze-pump-thaw cycles, was added and the resulting mixture was stirred at room temperature overnight. After that, the crude was dissolved in toluene (Tol) and passed through a short celite plug. The solvent was removed by vacuum distillation and the resulting dark solid was purified by column chromatography on silica gel using Tol/THF 40:1 as eluent and by size exclusion chromatography on Bio-beads using $CHCl_3$ as eluent. Upon recrystallization from methanol, **3** was obtained as a purple-pink solid (54 mg, 0.019 mmol). Yield: 91 %; **^{1}H-NMR** (300 MHz, THF-d_8): δ (ppm) = 9.37 (s, 3H, NH), 8.96 (s, 3H), 8.79 (d, $^3J_{H\text{-}H}$ = 8.28, 3H), 8.03 (dd, $^3J_{H\text{-}H}$ = 6.78 Hz, $^4J_{H\text{-}H=}$ 1.47 Hz, 3H), 7.85 (d, ^{3}JH-H = 8.73, 6H), 7.59 (d, $^3J_{H\text{-}H}$ = 8.64 Hz, 6H), 7.24 (s, 6H), 6.75 (d, $^3J_{H\text{-}H}$ = 8.64 Hz, 3H), 5.30 (d, $^3J_{H\text{-}H}$ = 8.91 Hz, 3H), 4.06 (t, 12H), 4.00 (t, 6H), 1.88–1.77 (m, 18H), 1.60–1.48 (m, 18H), 1.44–1.26 (m, 144 H), 1.07 (s, 9H), 0.89 (t, 27 H); **^{13}C-NMR** (75.5 MHz, THF d_8): δ (ppm) = 164.98, 153.00, 151.82, 141.65, 140.54, 132.08, 130.11, 125.31, 119.53, 115.55, 117.28, 106.55, 78.49, 72.75, 69.07, 31.94, 31.92, 30.71, 30.42, 29.81, 29.78, 29.72, 29.77, 29.62, 29.53, 29.46, 29.40, 29.37, 26.18, 22.60, 13.47; **^{11}B-NMR** (160.5 MHz, THF d_8): δ (ppm) = –14.43 (s, 1B); **MS** (MALDI-TOF, DCTB): m/z = 2860.0 [M]+. **HRLSI-MS** (DCTB + PPGNa 2000 + PPGNa 2700): m/z Calculated for [$C_{187}H_{268}BN_9O_{13}$]: 2861.0750; Found: 2861.0756; **UV/vis** (THF): λ_{max} (nm) (log ε (dm^3 mol^{-1} cm^{-1})) = 590 (8.7), 541 (3.0), 367 (5.1).

SubPc **4**

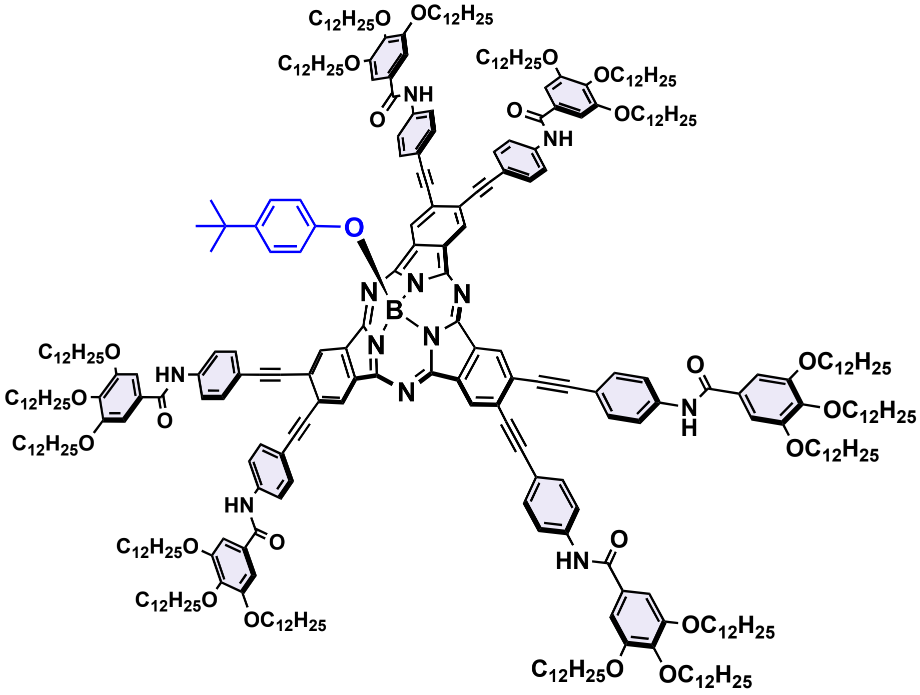

In a 25 mL Schlenk flask, equipped with a magnetic stirrer, **8** (17 mg, 0.013 mmol), $PdCl_2(PPh_3)_2$ (3.7 mg, 0.0053 mmol), CuI (2.0 mg, 0.011 mmol), and *N*–H (73 mg, 0.095 mmol) were placed under argon atmosphere. Then, 2.6 mL of a mixture of THF/NEt_3 5:1, which was deoxygenated via three freeze-pump-thaw cycles, was added and the resulting mixture was stirred first at 50 ºC for 30 min, and then at room temperature overnight. After that, the crude was dissolved in DCM and passed through a short celite plug. The solvent was removed by vacuum distillation and the resulting dark solid was purified by column chromatography on silica gel using DCM/MeOH 30:1 as eluent and by size exclusion chromatography on Bio-beads using $CHCl_3$ as eluent. Upon recrystallization from methanol, **4** was obtained as a green solid (44 mg, 0.0084 mmol). Yield: 64 %; **^{1}H-NMR** (300 MHz, THF d_8): δ (ppm) = 9.36 (s, 6H, NH), 8.87 (s, 6H), 7.76 (d, $^3J_{H\text{-}H}$ = 8.67 Hz, 12 H), 7.55 (d, $^3J_{H\text{-}H}$ = 8.55 Hz, 12H), 7.16 (s, 12H), 6.69 (d, $^3J_{H\text{-}H}$ = 8.7 Hz, 2H), 5.28 (d, $^3J_{H\text{-}H}$ = 8.64 Hz, 2H), 3.94 (t, 24H), 3.89 (t, 12H), 1.76–1.65 (m, 82H), 1.49–1.37 (m, 290H), 0.79 (t, 9H), 0.78 (t, 54H); **^{13}C-NMR** (75.5 MHz, THF d_8): δ (ppm) = 165.24, 153.06, 153.02, 152.01, 150.45, 142.91, 141.74, 140.52, 132.17, 129.90, 129.42, 126.97, 125.39, 119.80, 117.72, 117.58, 106.57, 96.23, 87.95, 72.79, 69.08, 66.78, 66.73, 66.61, 66.43, 66.32, 66.25, 66.08, 31.94, 29.73, 29.71, 29.70, 24.67, 24.51, 24.35, 24.19, 24.03, 22.61, 13.50; **^{11}B-NMR** (160.5 MHz, THF d_8): δ (ppm) = –14.01 (s, 1B); **MS** (MALDI-TOF, DCTB): m/z = 5176.9 [M]+. **HRLSI-MS** (DCTB + PMMANa 4300 + NaI): m/z Calculated for $[C_{340}H_{511}BN_{12}NaO_{25}]$: 5199.9181; Found: 5199.9157; **UV/vis** (THF): λ_{max} (nm) (log ε(dm^3 mol^{-1} cm^{-1}))) = 620 (9.7), 564 (2.9), 390 (6.5).

3. NMR spectra

SubPc **1-*Rac***

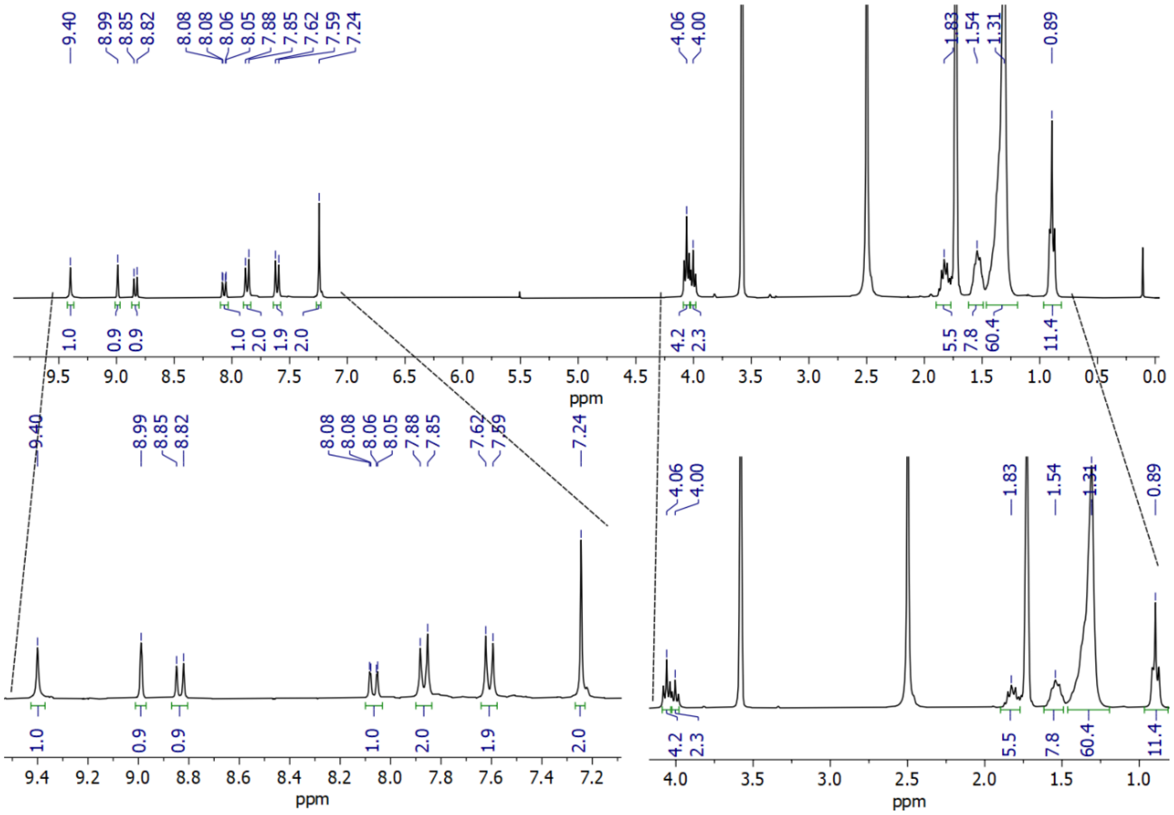

Figure S3.1. ^{1}H-NMR spectrum (THF-d_8) of **1**

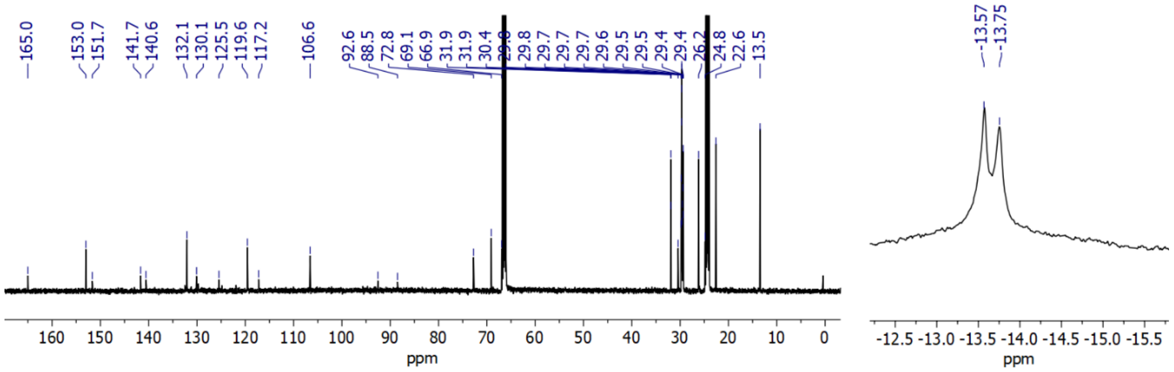

Figure S3.2. ^{13}C-NMR (left) and ^{11}B-NMR (right) spectra (THF-d_8) of **1**

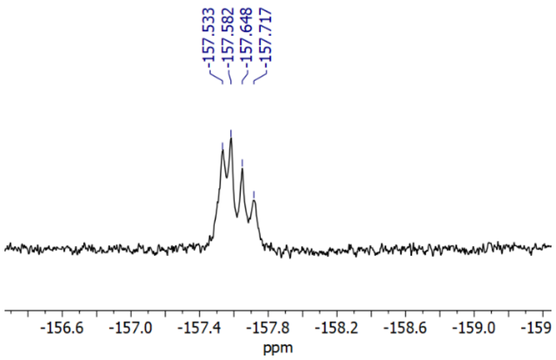

Figure S3.3. ^{19}F-NMR spectrum (THF-d_8) of **1**

SubPc **1-*M***

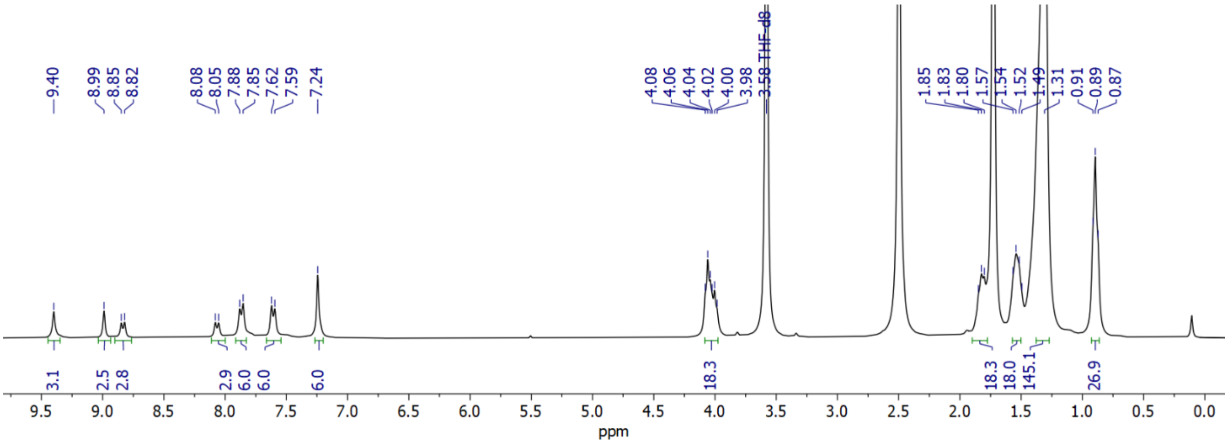

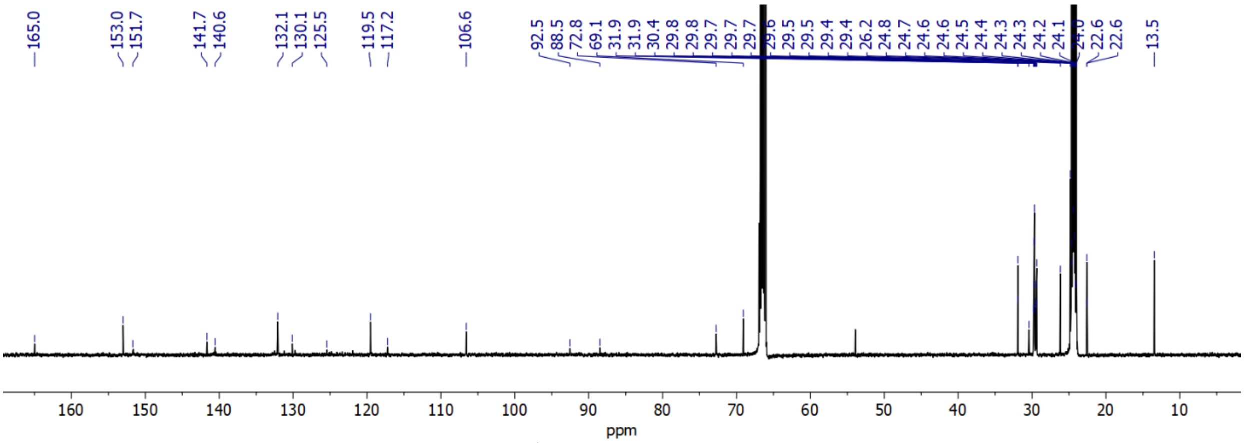

Figure S3.4. ^{1}H-NMR spectrum (THF-d_8) of **1-*M***

Figure S3.5. ^{13}C-NMR spectrum (THF-d_8) of **1-*M***

Figure S3.6. ^{11}B-NMR (left) and ^{19}F-NMR (right) spectra (THF-d_8) of **1-*M***

SubPc **1-*P***

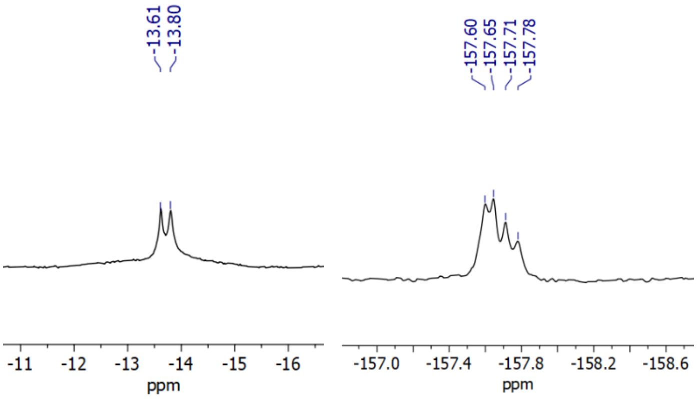

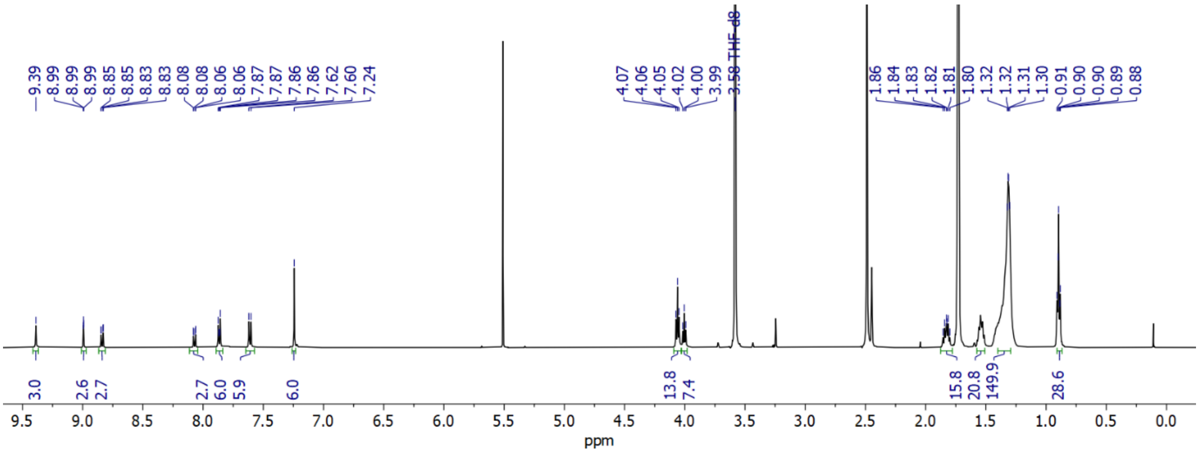

Figure S3.7. ^{1}H-NMR spectrum (THF-d_8) of **1-*P***

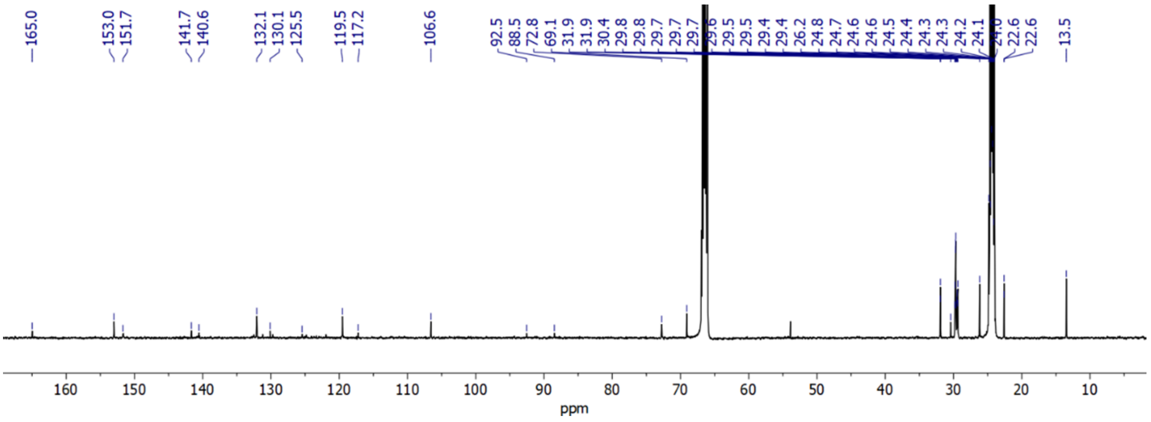

Figure S3.8. ^{13}C-NMR spectrum (THF-d_8) of **1-*P***

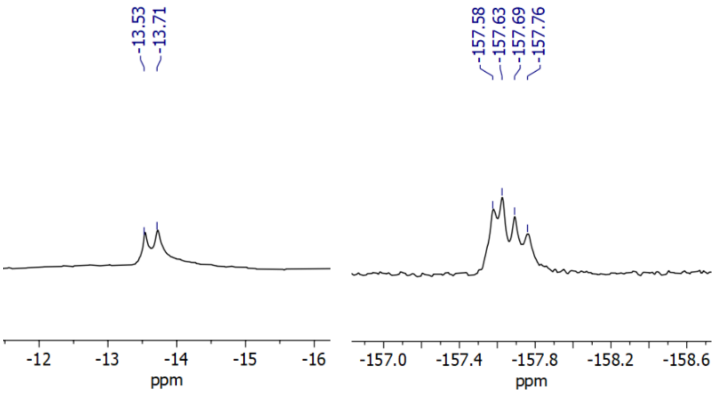

Figure S3.9. ^{11}B-NMR (left) and ^{19}F-NMR (right) spectra (THF-d_8) of **1-*P***

SubPc **2**

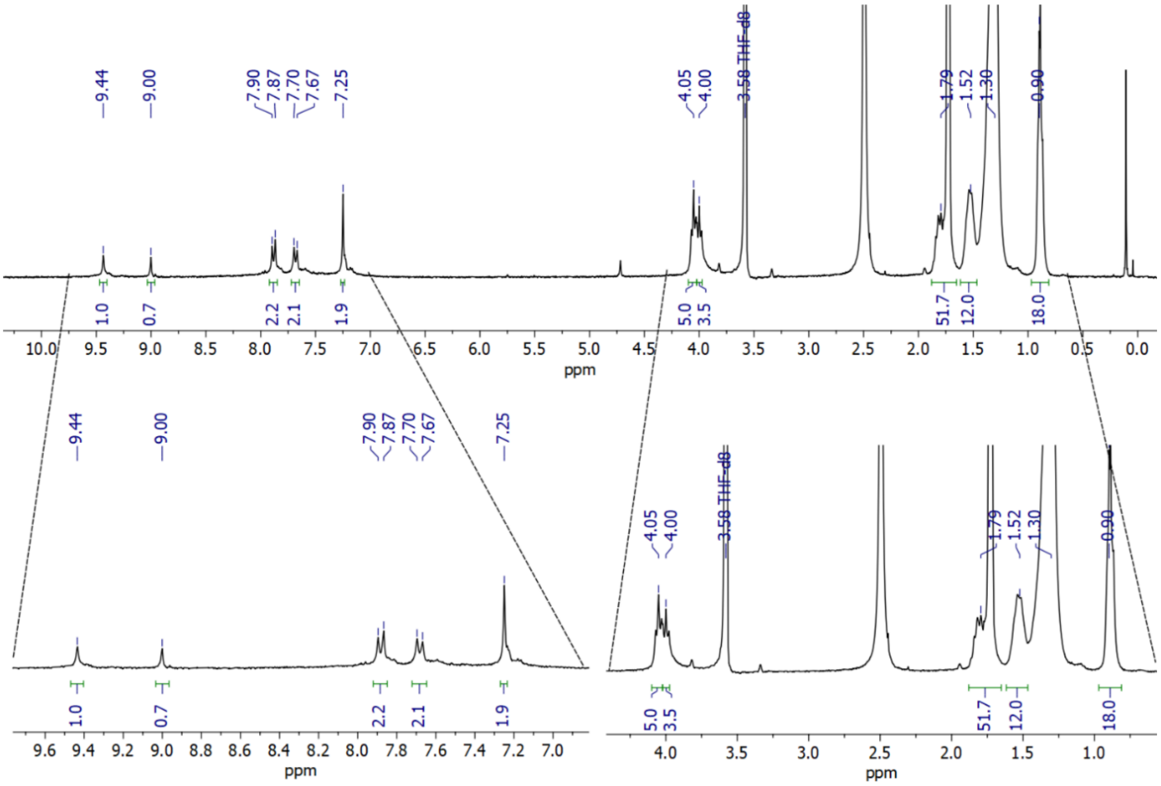

Figure S3.10. ^{1}H-NMR spectrum (THF-d_8) of **2**

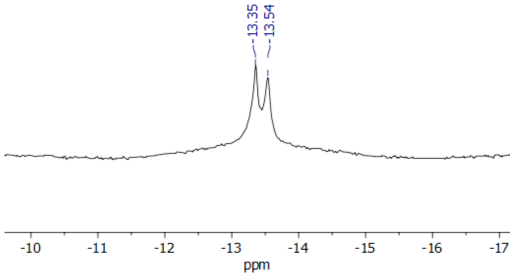

Figure S3.11. ^{11}B-NMR (right) spectra (THF-d_8) of **2**

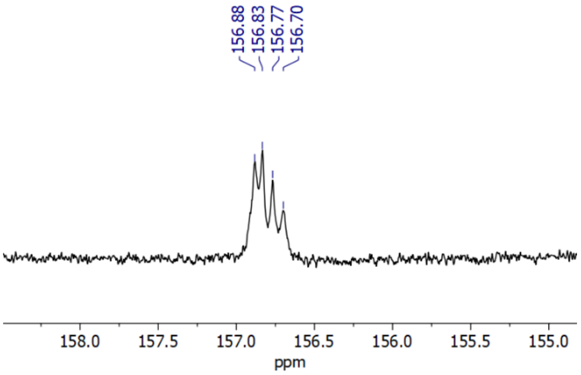

Figure S3.12. ^{19}F-NMR spectrum (THF-d_8) of **2**

SubPc **3**

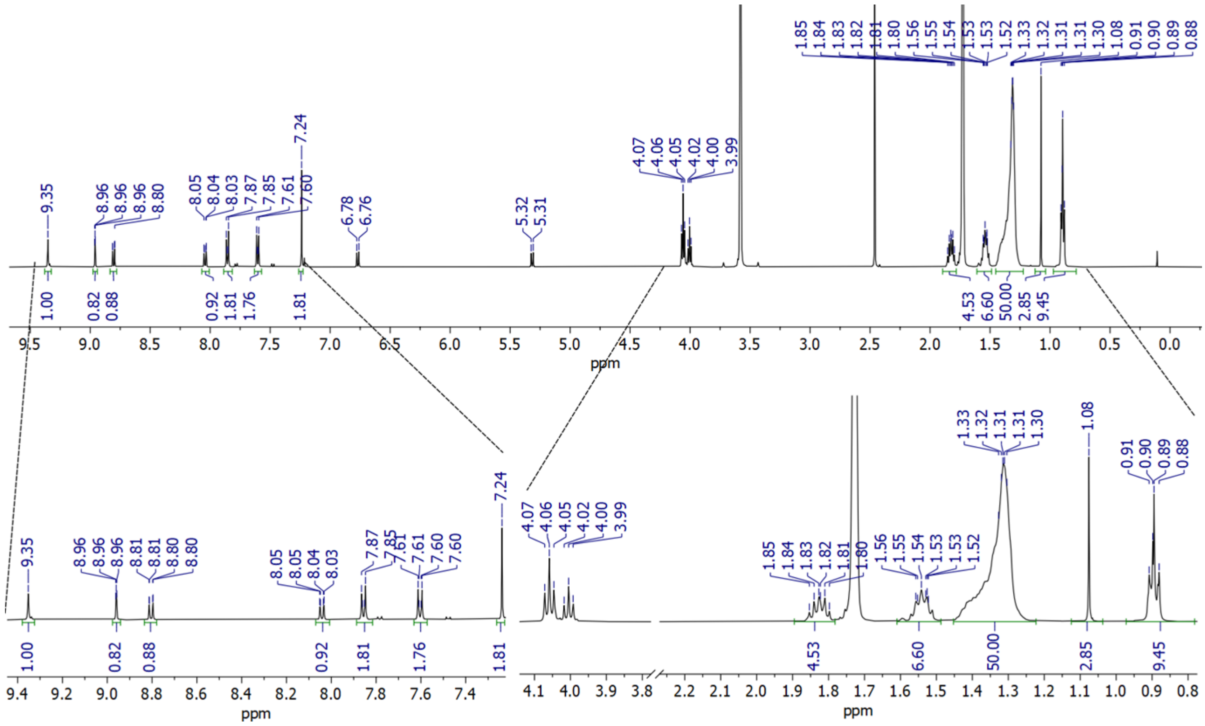

Figure S3.13. ^{1}H-NMR spectrum (THF-d_8) of **3**

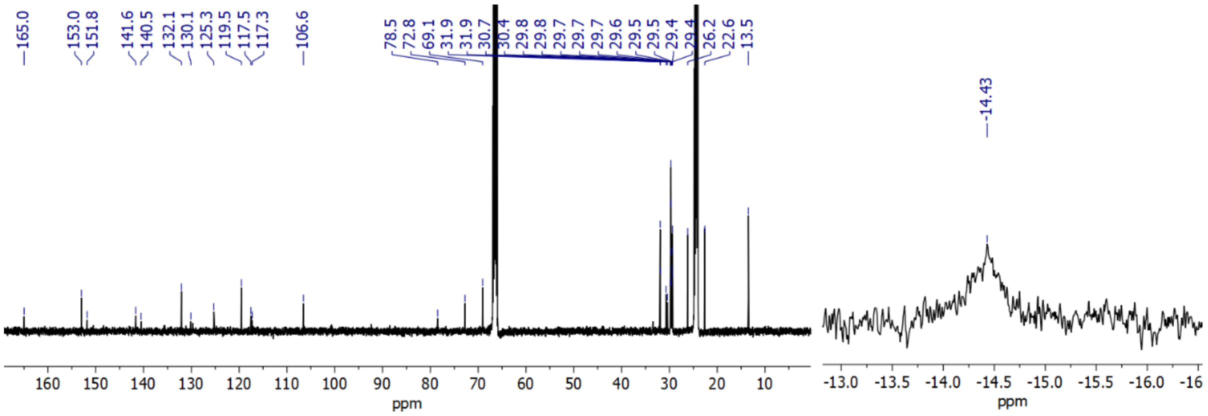

Figure S3.14. ^{13}C-NMR (left) and ^{11}B-NMR (right) spectra (THF-d_8) of **3**

SubPc **4**

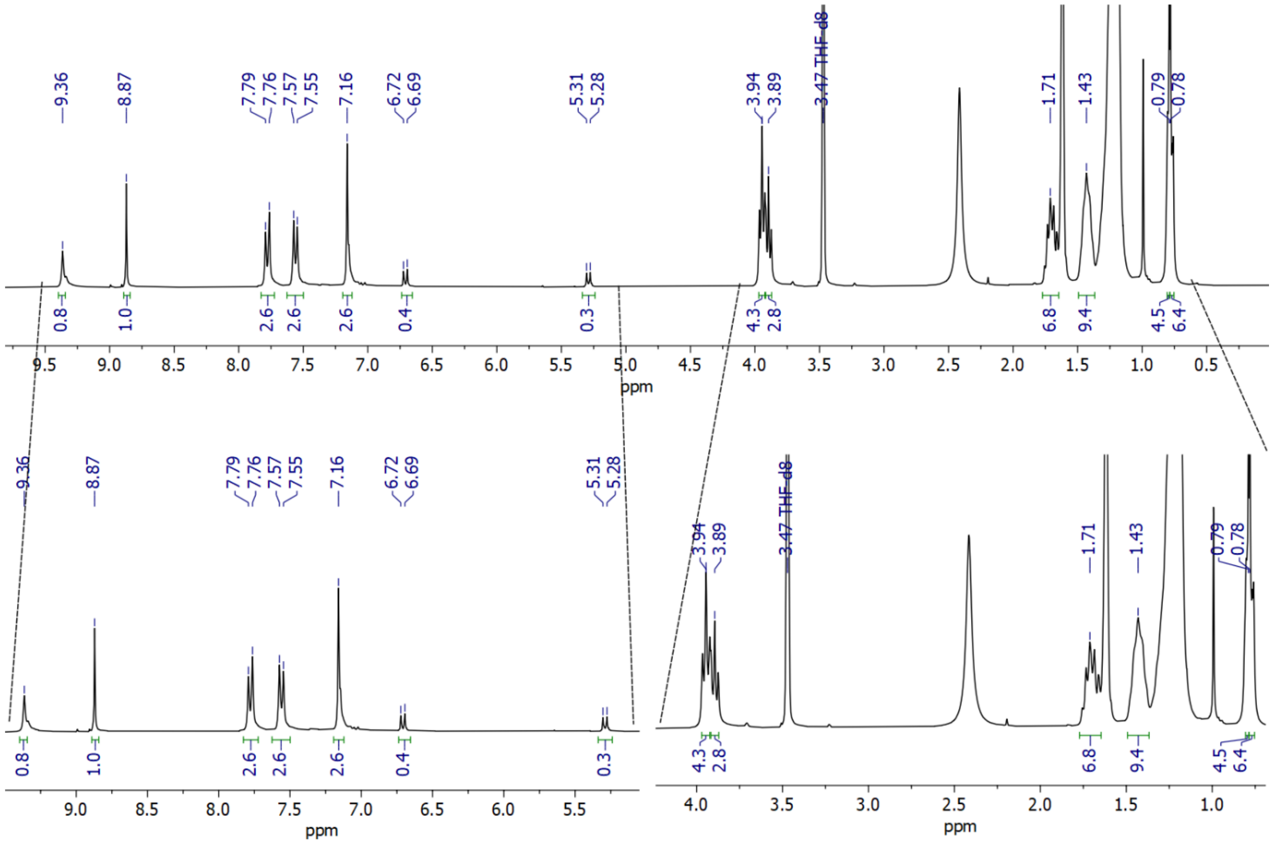

Figure S3.15. ^{1}H-NMR spectrum (THF-d_8) of **4**

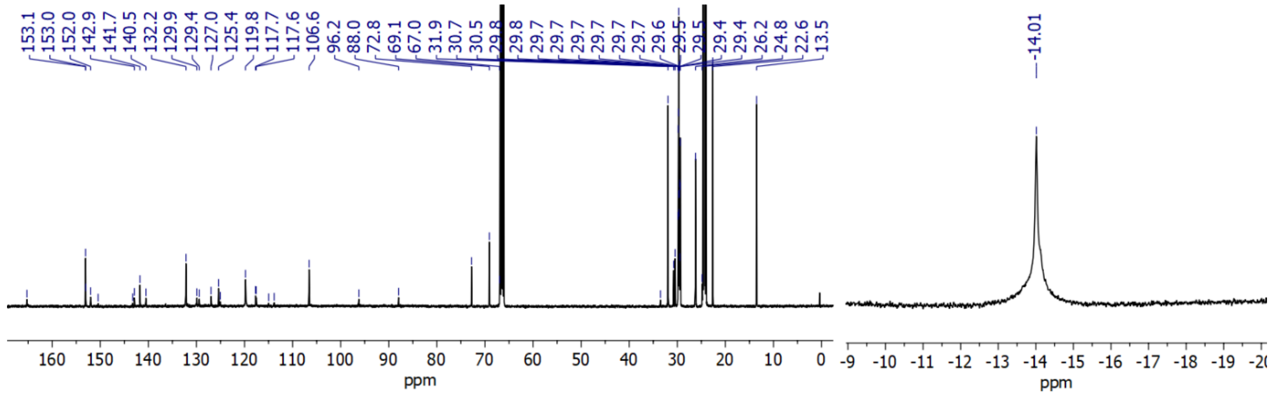

Figure S3.16. ^{13}C-NMR (left) and ^{11}B-NMR (right) spectra (THF-d_8) of **4**

4. Mass spectra

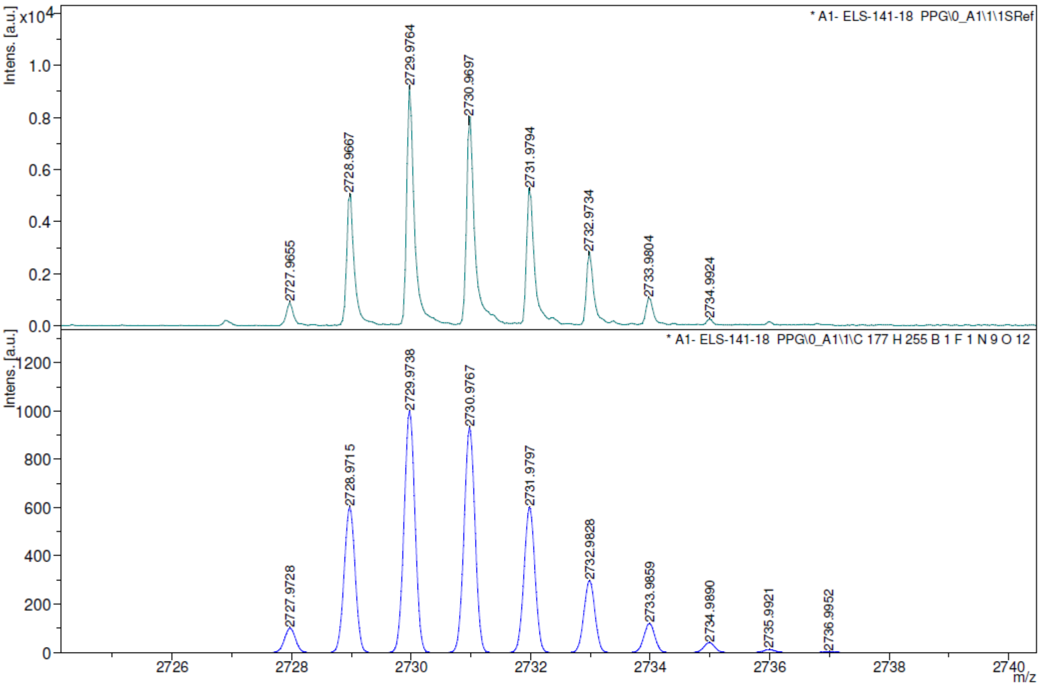

Figure S4.1. MALDI-TOF mass spectrum of **1-*Rac***

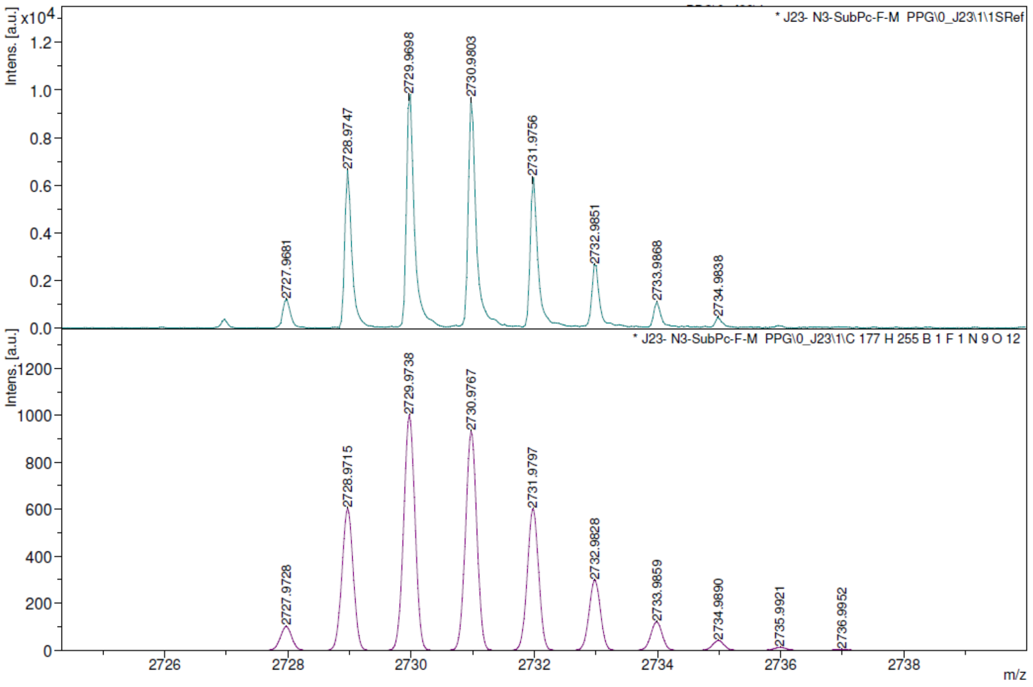

Figure S4.2. MALDI-TOF mass spectrum of **1-*M***

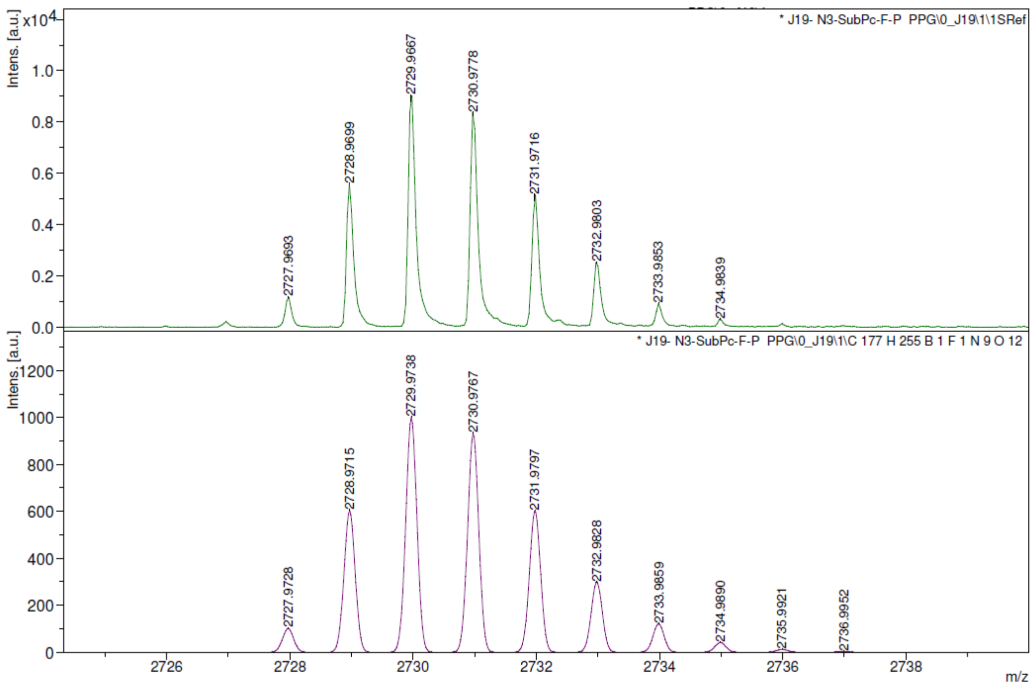

Figure S4.3. MALDI-TOF mass spectrum of **1-*P***

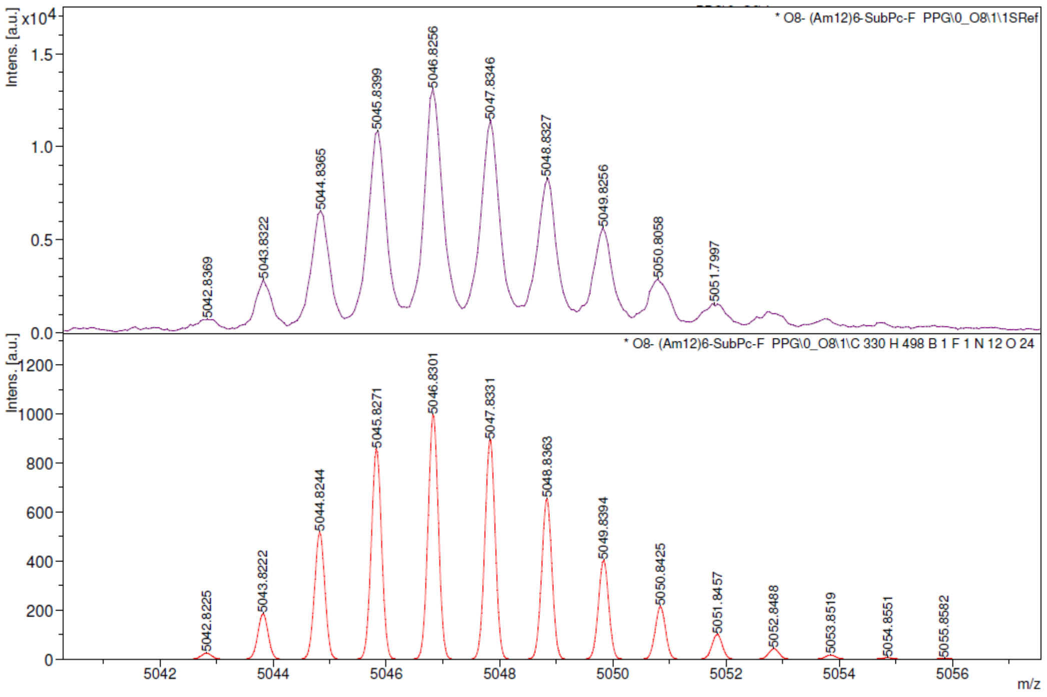

Figure S4.4. MALDI-TOF mass spectrum of **2**

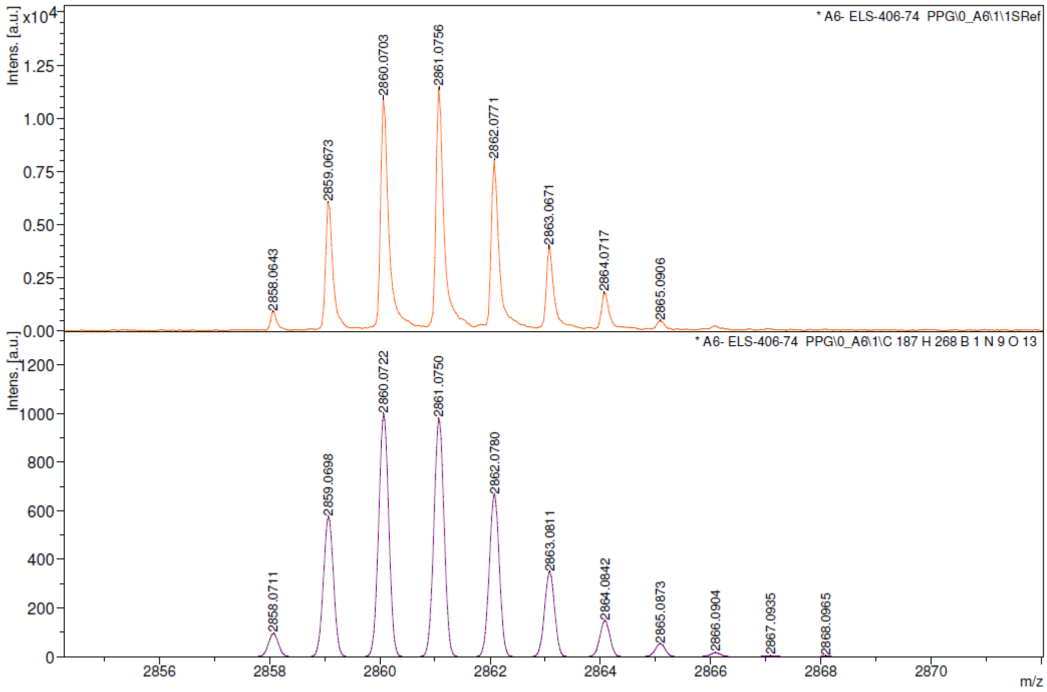

Figure S4.5. MALDI-TOF mass spectrum of **3**

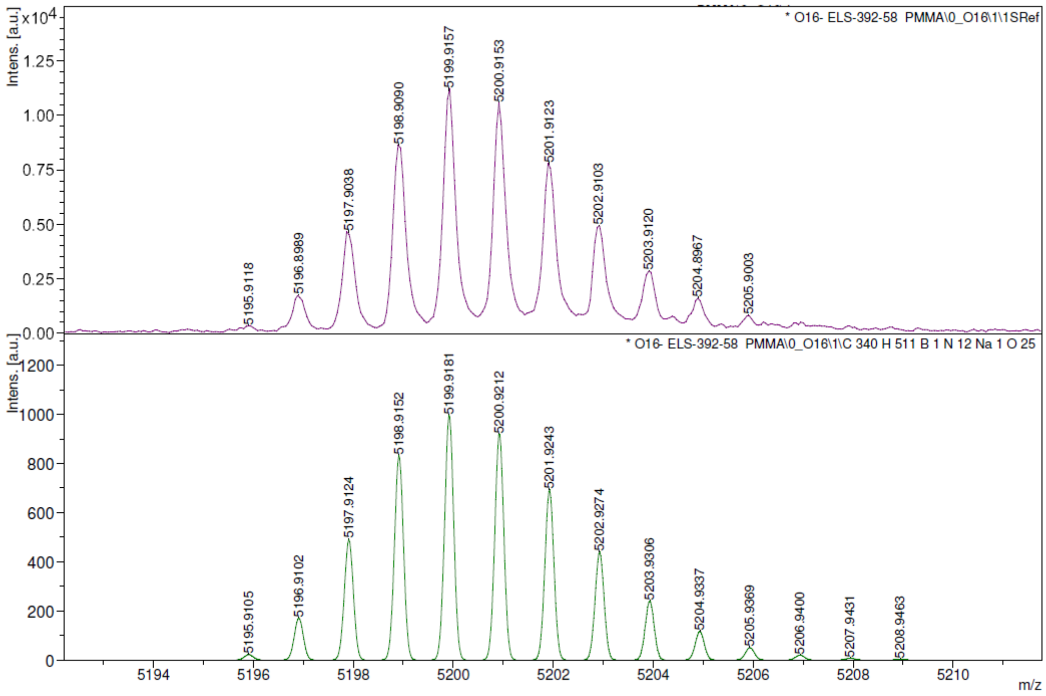

Figure S4.6. MALDI-TOF mass spectrum of **4**

5. Additional aggregation studies

5.1. NMR Studies

The aggregation capability of **1-*Rac*** and **2** was further assessed by NMR spectroscopy. To this end, ^{1}H NMR spectra of both compounds in TCE were recorded upon increasing the temperature (258 K to 368 K). We employed TCE since in MCH at this high concentration (0.5 mM) both compounds exist in a fully aggregated state (NMR silent). As shown in Figure S5.1, both VT NMR spectra reveal a broadening and shifting of the signals corresponding to the SubPc aromatic core, suggesting head-to-tail π-π stacking.

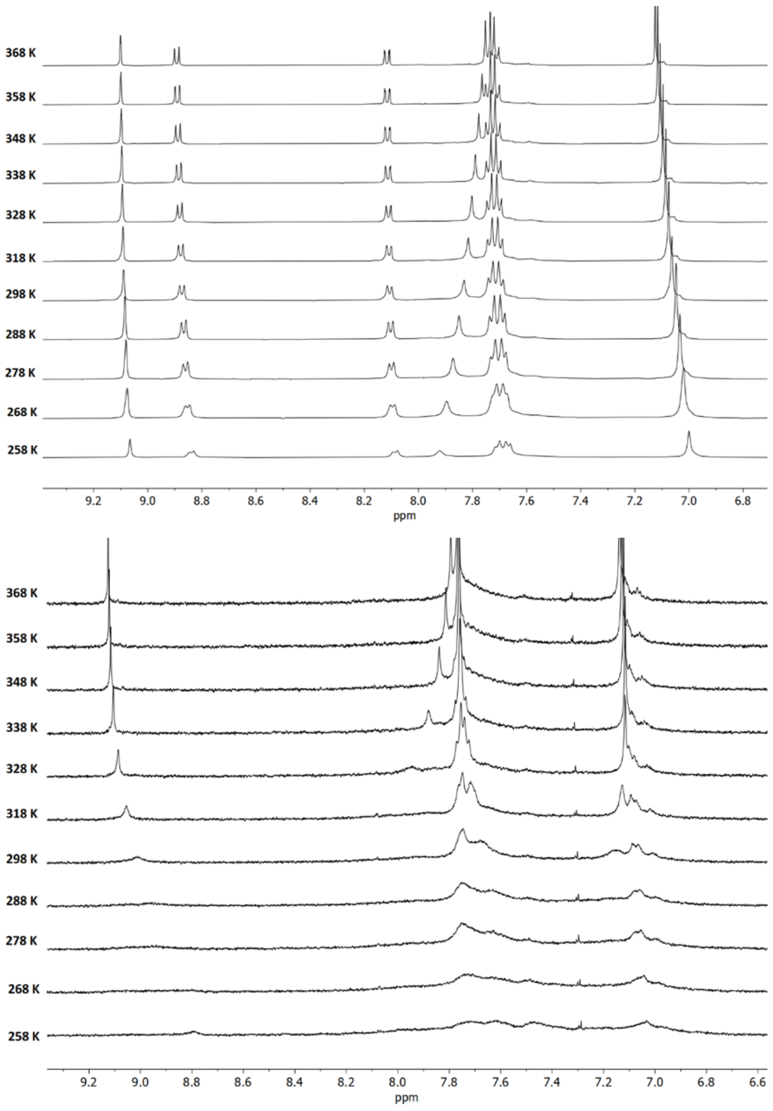

Figure S5.1. Changes in the aromatic region of the ^{1}H NMR spectra of **1-*Rac*** (top) and **2** (bottom) in $CDCl_2CDCl_2$ as a function of temperature at 0.5 mM.

5.2. Nucleation-elongation model for cooperative supramolecular polymerization from variable temperature experiments

The model developed by Ten Eikelder, Markvoort, Meijer and co-workers[7] extends the nucleation-elongation equilibrium models designed to describe the growth of supramolecular homopolymers to the case of two monomers and aggregate types and can be applied to symmetric or non-symmetric supramolecular copolymerizations.

A cooperative supramolecular mechanism can be divided into a nucleation (nucleus size of 2) and an elongation phase. The values of T_e, ΔH°_n, ΔH°, and ΔS° can be obtained from a non-linear least-square analysis of the experimental melting curves and the equilibrium constants associated to the nucleation (K_n) and elongation (K_e) phases as well as the cooperativity factor (σ) can be calculated using equations 1, 2, and 3, respectively (see Table 1 in the text).

$$K_n = e^{\left(\frac{-(\Delta H^\circ - \Delta H_n^\circ - T\Delta S^\circ)}{RT}\right)} \quad (1)$$

$$K_e = e^{\left(\frac{-(\Delta H^\circ - T\Delta S^\circ)}{RT}\right)} \quad (2)$$

$$\sigma = \frac{K_n}{K_e} = e^{\left(\frac{\Delta H_n^\circ}{RT}\right)} \quad (3)$$

5.3. Nucleation-elongation model for cooperative supramolecular polymerizations from good solvent experiments

In this equilibrium model, the monomer addition steps in the nucleation regime are described by an equilibrium nucleation constant K_n with a cooperative parameter (σ):[8]

$$\sigma = \frac{K_n}{K_e} < 1 \quad (4)$$

The elongation equilibrium constant K_e is defined via:

$$K_e = e^{\left(\frac{-\Delta G^{\circ\prime}}{RT}\right)} \quad (5)$$

where $\Delta G^{\circ\prime}$ is the Gibbs free energy gain upon monomer addition, R the gas constant, and T the temperature. According to denaturation models, the Gibbs free energy is assumed to be linearly dependent on the volume fraction of the good solvent (χ_{THF}):

$$\Delta G^{\circ\prime} = \Delta G^\circ + m \cdot \chi_{THF} \quad (6)$$

ΔG° represents the Gibbs free energy gain upon monomer addition in the pure solvent (MCH) and the dependence of $\Delta G^{\circ\prime}$ on χ_{THF} is described by the m parameter, which characterizes the ability of the good solvent to associate with the monomer thereby destabilizing the supramolecular aggregated species.

5.4. Aggregation studies of 1-*P*

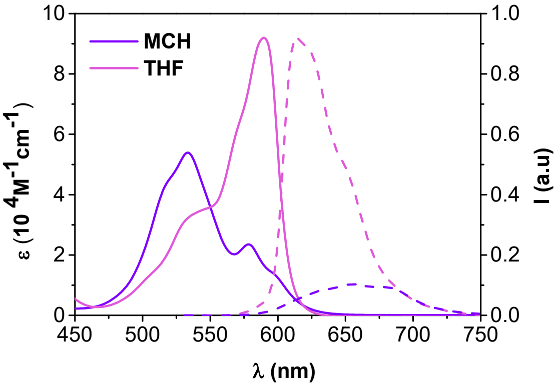

Figure S5.2. Absorption (solid lines) and emission (dashed lines λ_{exc} = 545 nm) spectrum of **1-*P*** in THF and MCH ([SubPc]= 6.5 × 10^{-6} M).

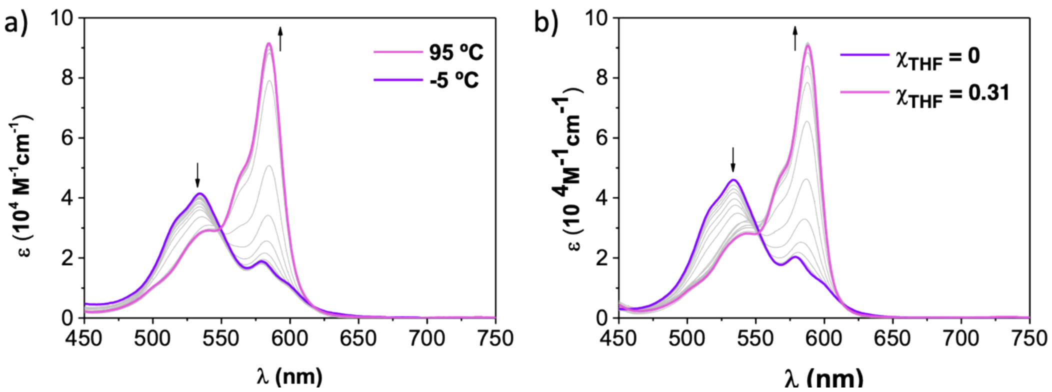

Figure S5.3. Absorption changes recorded for **1-*P*** as a function of a) temperature at 6.5 x 10^{-6} M in MCH and b) solvent (THF/MCH) composition at 6.5 x 10^{-6} M.

5.5. Fits to a nucleation−elongation model at different concentrations

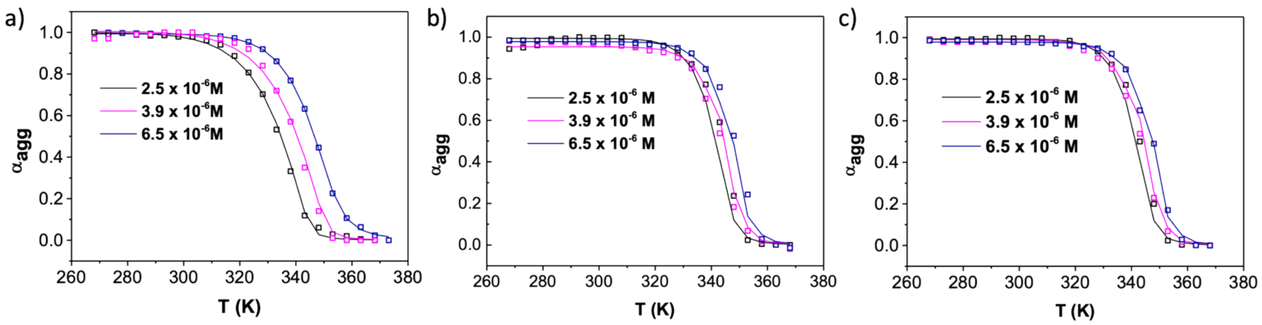

Figure S5.4. Evolution of the aggregation degree (α_{agg} versus T) and the corresponding global fit (solid line) to a nucleation−elongation model at different concentrations of a) **1-*Rac***, b) **1-*M*** and c) **1-*P***.

Table S5.1. Thermodynamic parameters calculated upon: a) polymerization of **1-*P*** by decreasing temperature in MCH, and b) depolymerization of **1-*P*** in MCH:THF mixtures by increasing the volume fraction of THF (χ_{THF}).

		K_n (M^{-1})	K_e (M^{-1})	T_e (K)	σ	$\Delta H°$ (kJ mol^{-1})	$\Delta S°$ (J mol^{-1} K^{-1})	$\Delta H_n°$ (kJ mol^{-1})	m (kJ mol^{-1})	$\Delta G°$ (kJ mol^{-1})
VT	**1-*Rac***	1.8×10^3	3.0×10^5	349.0 ± 0.7	0.006	–91.0 ± 1.0	–119.0 ± 3.0	–10.6 ± 0.9		
VT	**1-*M***	8.2×10^3	2.2×10^5	348.3 ± 0.3	0.037	–160.0 ± 5.8	–339.0 ± 1.0	–9.3 ± 0.8		
GS	**1-*Rac***				0.004				115.0 ± 7.0	–70.2 ± 1.1
GS	**1-*M***				0.041				98.1 ± 9.7	–45.2 ± 0.9

5.6. CD aggregation studies

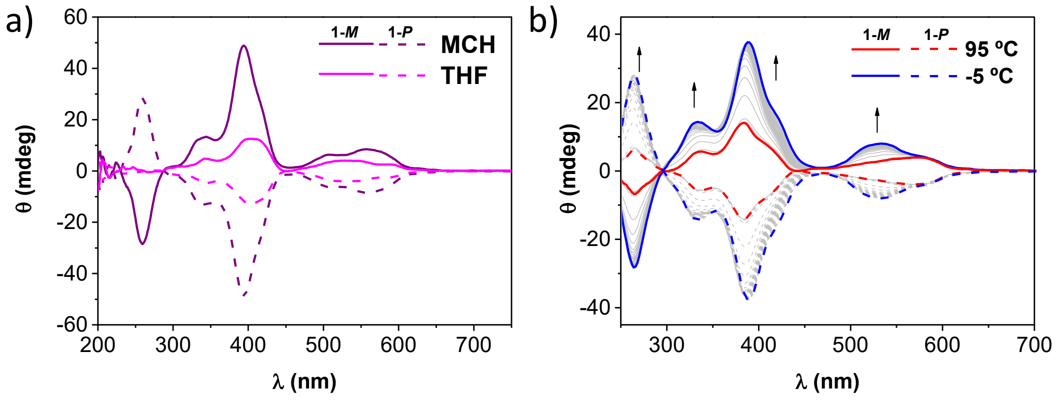

Figure S5.5. a) CD spectrum of **1-*M*** and **1-*P*** in MCH and THF (3.2×10^{-5} M). b) CD changes as a function of temperature in MCH (3.2×10^{-5} M).

6. AFM Measurements

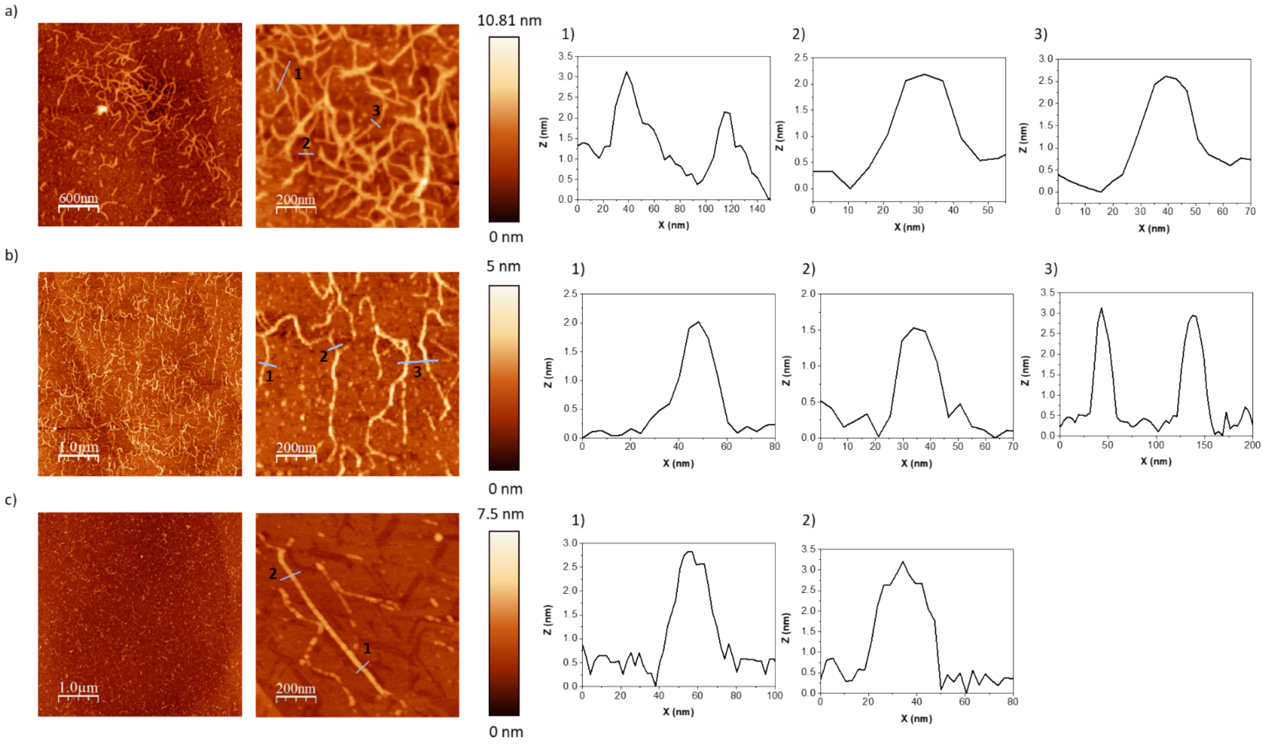

Figure S6.1. AFM images of a) drop-casted **2**, b) **1-*Rac***, and c) **1-*M*** onto HOPG in MCH (3.9×10^{-6} M) in MCH. eight profiles along the grey lines marked in the AFM images are represented.

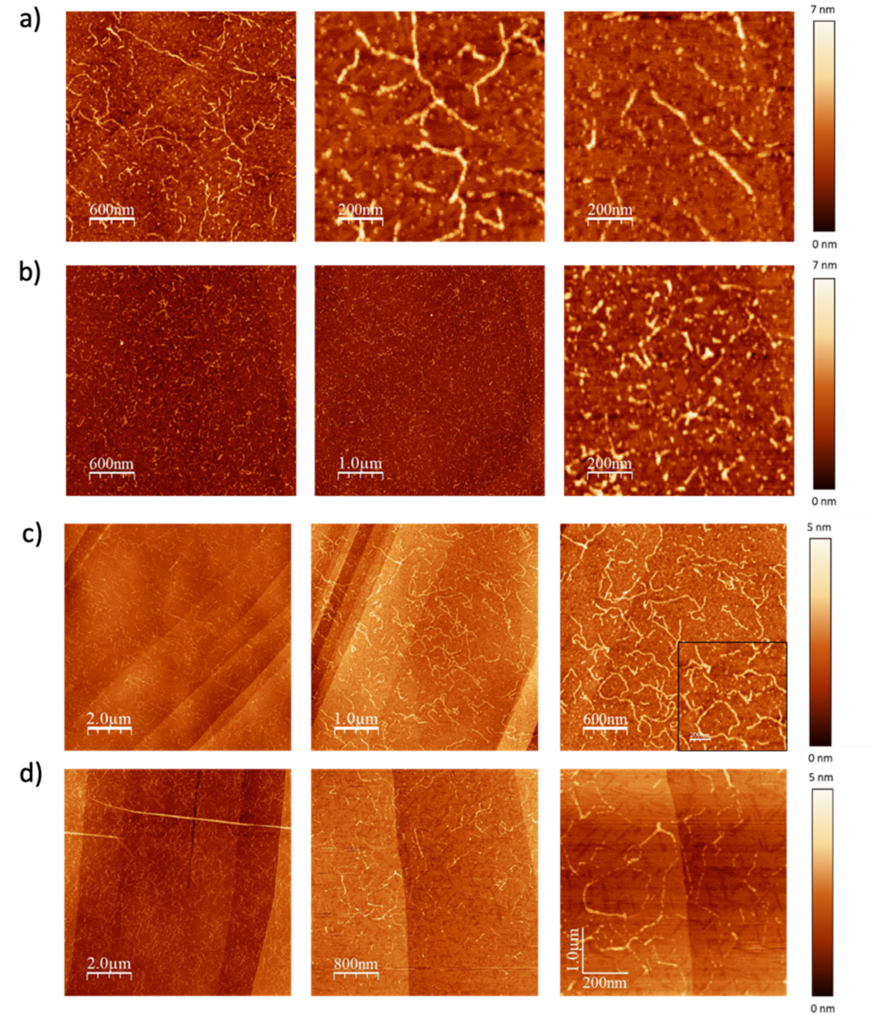

Figure S6.2. Additional AFM images at different concentrations (1 × 10^{-6} M for a and b; and 5 × 10^{-6} M for c and d) of a,c) **1-*Rac*** and b,d) **1-*M*** drop-casted onto HOPG in MCH.

7. Computational studies

7.1. Force Field Parametrization

The quantum mechanically derived force field (QMDFF) used to perform the molecular dynamics calculations on SubPcs **1** and **2** was obtained by parametrizing the molecular fragments showed in **Figure S7.1**, which were calculated at the density functional theory (DFT) B3LYP/cc-PVDZ level, and transferring the parameters to the full structure.

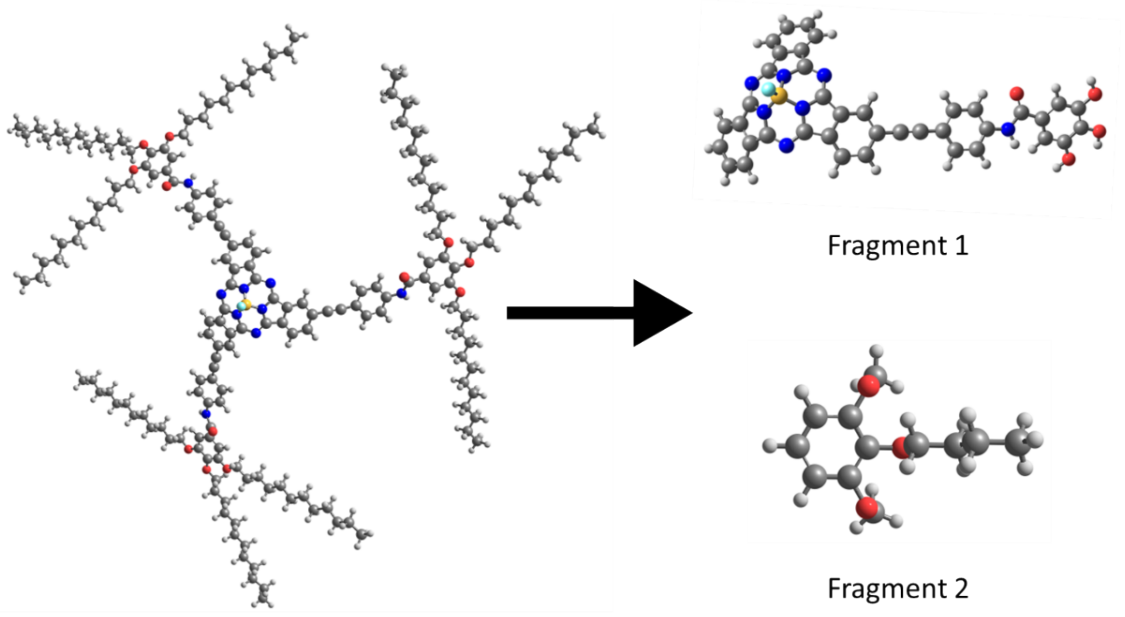

Figure S7.1. Chemical structure of SubPc **1-*P*** (left) and molecular fragments used to obtain the FF parameters of **1** and **2** (right).

Fragment 1 includes the SubPc triisoindole core and one peripheral 3,4,5-tris(metoxy)-*N*-(4-ethynylphenyl)benzamide substituent. It incorporates the terminal aryl group just to properly represent the amide group parameters, which are indeed affected by the aromatic moiety. Fragment 2 is a simplied model of the terminal 3,4,5-tris(dodecyloxy)phenyl groups. To build this fragment, all atoms from position γ in the alkyl chain to the end of the dodecyloxy group were considered identical, except for the last one, which is a methyl group. Therefore, a butyl chain is long enough. The parameters obtained for this central chain were transferred to those in positions 3 and 5, except for the rotation of the methyl group with respect to the aromatic ring, which is indeed specific for each substituent. This was done to reduce the computational cost of the DFT calculations. Finally, due to the molecular symmetry, the parameters obtained for the 3,4,5-tris(dodecyloxy)-*N*-(4-ethynylphenyl)benzamide substituent (*N* group in Figure in the main text) were replicated for all the 3 (in **1**) or 6 (in **2**) substituents present in the full systems.

In all cases, bonded interactions were obtained by following the protocol implemented in the JOYCE code.[9] The protocol consists on a fitting of the quantum mechanical Hessian to obtain the parameters of rigid coordinates whose energy profile adjusts well to a harmonic profile (bond stretching, bond angles, and out-of-plane dihedral of aromatic cycles), whereas for flexible coordinates the energy profile of each dihedral was computed with DFT and reproduced by a Fourier series. The atom type selection is shown in **Figure S7.2**. For nonbonded interactions, point charges were computed with the electrostatic potential (ESP) method,[10] whereas van der Waals interactions were described by a Lennard-Jones potential with parameters taken from the OPLS database,[11] except for B and F which were taken from the literature,[12] as shown in **Table S7.1**. The interaction terms were computed as the geometric average of the atomic parameters involved in each particular pair.

The details of each fragment parametrization are described here following.

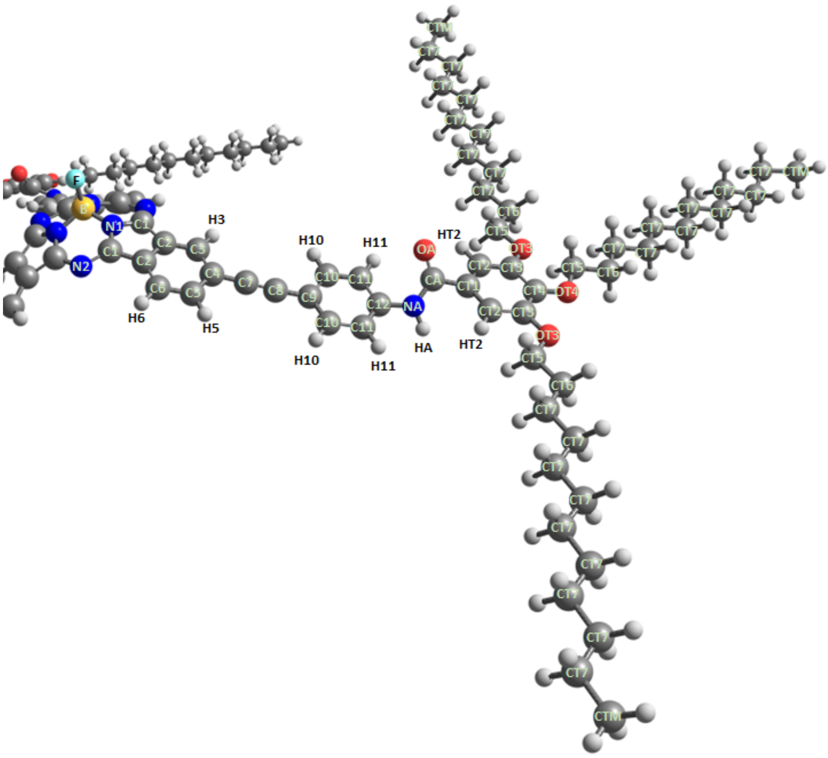

Figure S7.2. Atom type selection. Only one substituent of the SubPc core is emphasized, the other two being equivalent by molecular symmetry. In the dodecyloxy groups, the labeling of the hydrogen atoms (not shown for simplicity) is analogous to that used for the carbon atoms they are bonded to, *i.e.*, HT5, HT6, HT7, and HTM respectively denote hydrogens attached to CT5, CT6, CT7, and CTM.

Table S7.1. Nonbonded Lennard-Jones parameters for all atom types. The expression of the Lennard-Jones potential between atoms i and j separated by distance r_{ij} is

$$V_{LJ}(r_{ij}) = 4\varepsilon_{ij}\left[\left(\frac{\sigma_{ij}}{r_{ij}}\right)^{12} - \left(\frac{\sigma_{ij}}{r_{ij}}\right)^{6}\right].$$

Atom	σ (nm)	ϵ (kJ·mol^{-1})
B	0.35814	0.39748
F	0.29400	0.255224
N (N1, N2 and NA)	0.32500	0.711280
Aromatic C (C1-C6, C9-C12, CT1-CT4)	0.35500	0.29288
Alkyne C (C7-C8)	0.33000	0.56484
Aromatic H (H3, H5, H6, H10-H11, HT2)	0.24200	0.12552
Amide C (CA)	0.37500	0.43932
Amide O (OA)	0.29600	0.87864
Amide H (HA)	0.00000	0.00000
OT3, OT4	0.29000	0.58576
Aliphatic C (CT5-CT7, CTM)	0.35000	0.276144
Aliphatic H (HT5-HT7, HTM)	0.25000	0.12552

Parametrization of Fragment 1

Fragment 1 provides all the parameters for the SubPc core, the substituent, and the amide group. In addition, the energy profiles of the four flexible dihedrals (δ_1-δ_4) are shown in **Figure S7.3**. These dihedrals include the rotation around the ethynyl group (δ_1) and those involving the amide group (δ_2-δ_4). Specifically, δ_1 is represented by the C5−C7−C8−C10 dihedral, δ_2 by C11−C12−NA−CA, δ_3 by C12−NA−CA−CT1, and δ_4 by NA−CA−CT1−CT2. The rotation energy profiles were fitted to DFT B3LYP/cc-PVDZ calculations.

The total standard deviation for rigid coordinates between the DFT Hessian and the FF parametrization accounts to 4.11 × 10^{-3} kJ mol^{-1}. The molecular structure of Fragment 1 was optimized with the FF and the resulting geometrical parameters were compared with those of the DFT minimum-energy geometry, providing a root-mean square deviation of 0.000 Å, 0.006º, and 0.678º for bond length, bond angles, and dihedrals, respectively. The displacements of all atoms between the FF and the DFT minima amounts to only 0.085 Å. The FF results therefore show a very good fitting of the DFT structure, considering the large number of atoms in Fragment 1 (73), and validate the accuracy of the parameterization obtained. For the flexible coordinates shown in Figure 3, the fitting is also in very good agreement with DFT results.

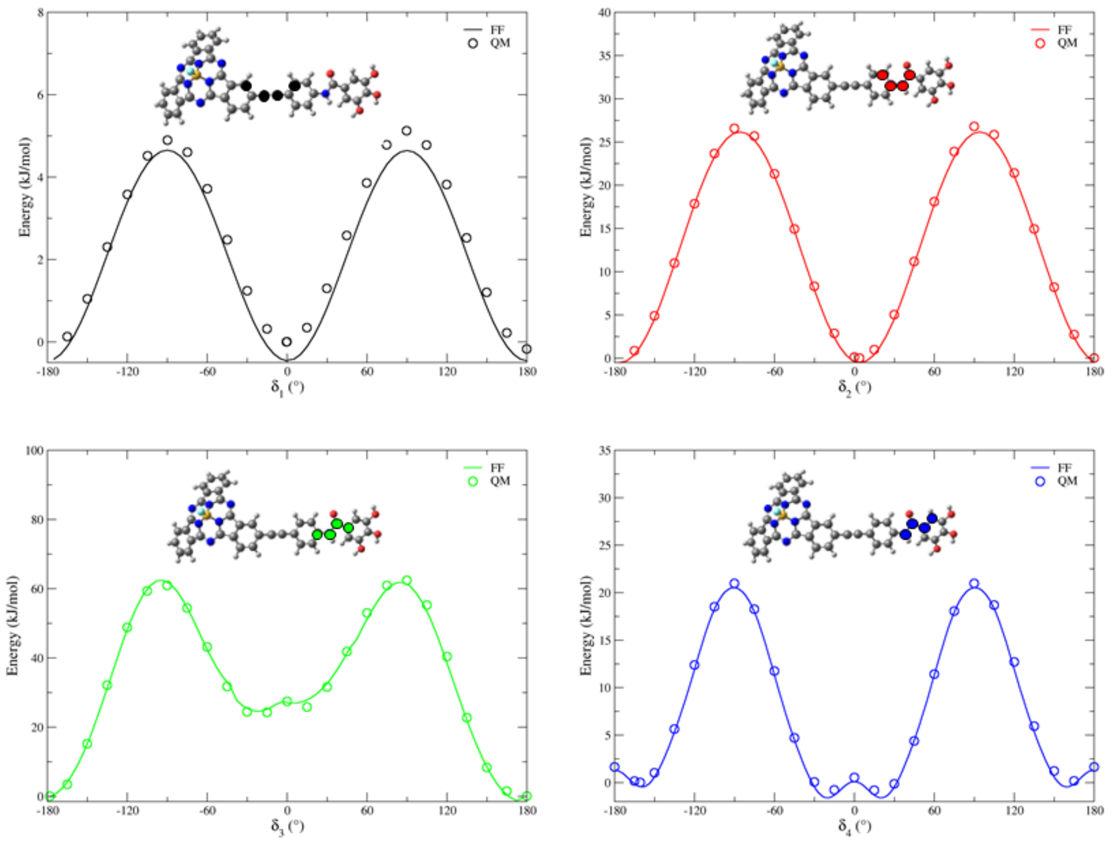

Figure S7.3. Flexible dihedrals δ_1-δ_4 defined on Fragment 1 and their energy profiles computed with DFT (circles) or fitted (lines). The colored spheres in the structural model represent the atoms involved in each specific dihedral. Note that the energy scale on the *y* axis is different for each panel.

Parametrization of Fragment 2

Fragment 2 describes the 3,4,5-tris(dodecyloxy)phenyl groups end-capping the SubPc substituents. Another six flexible dihedrals (δ_5-δ_{10}) were defined and their energy profile fitted to DFT calculations are shown in **Figure S7.4.** The dihedrals involve the relative rotation around the C–O bonds (δ_5-δ_7), and the dihedrals related to the alkyl chain group. For simplicity, all dihedrals involving four alkyl carbon atoms were considered identical. Therefore, only three additional dihedrals (δ_8-δ_{10}) were parametrized. In particular, δ_5 and δ_7 are equivalent and represent the CT2−CT3−OT3−CT5 dihedrals, δ_6 corresponds to the CT3−CT4−OT4−CT5 dihedral, and δ_8 to δ_{10} are the CT4−OT4−CT5−CT6, OT4−CT5−CT6−CT7, and CT5−CT6−CT7−CT7 dihedrals, respectively.

The total standard deviation for rigid coordinates between the DFT Hessian and the FF parametrization accounts to 7.87×10^{-3} kJ mol^{-1}. The molecular structure of Fragment 2 was optimized with the FF and the resulting geometric parameters were compared with those of the DFT minimum-energy geometry, providing a root-mean square deviation of 0.000 Å, 0.033º, and 0.463º for bond lengths, bond angles, and dihedrals, respectively. The displacements of all atoms between the FF and the DFT minima amounts to 0.028 Å, again evidencing a very good fitting to the DFT structure.

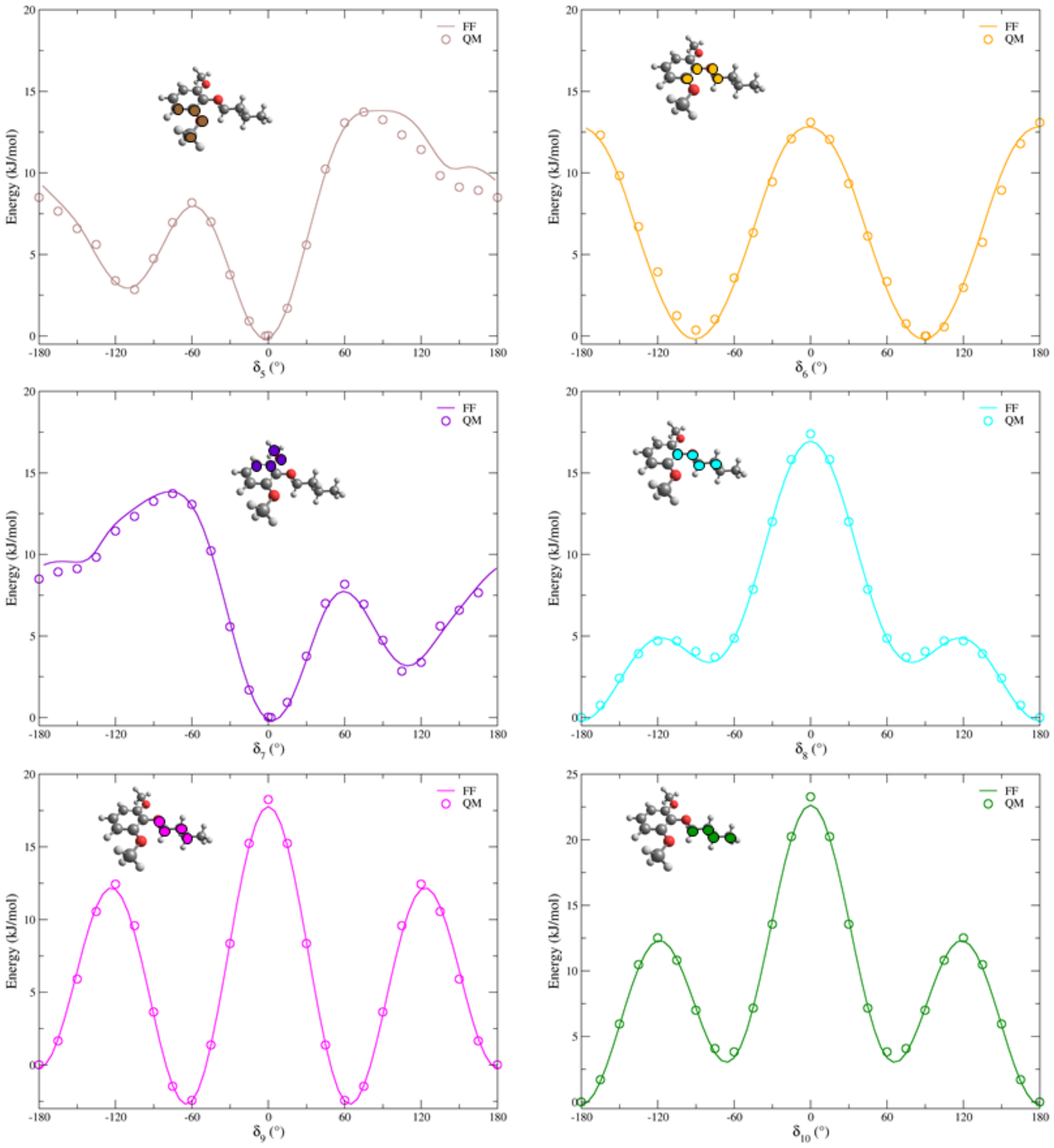

Figure S7.4. Flexible dihedrals δ_5-δ_{10} defined on Fragment 2 and their energy profiles computed with DFT (circles) or fitted (lines). The colored spheres on the structural model represent the atoms involved in each specific dihedral. Note that the energy scale on the *y* axis may differ for each panel.

7.2. Computational Details

Molecular dynamics (MD) simulations in methylcyclohexane (MCH) as solvent were performed with GROMACS 2021.3 using periodic boundary conditions.[13] For SubPcs, we used the quantum-mechanically derived force field (QMDFF) presented in this work with the atom type selection discussed in the previous section. The corresponding FF of the enantiomer of **1** was obtained by simply flipping the sign of the equilibrium dihedral angle for rigid out-of-plane internal coordinates. To simulate the solvent MCH, the FF was generated with the PolyParGen tool,[14] and a compressibility of 11.49×10^{-5} bar^{-1} was used during the simulations. The initial structures used for monomers, dimers, and octamers were centered in a box large enough that the minimum distance between the box boundaries and the closest atom was of at least of 1.0 nm to avoid spurious interactions due to the periodic boundary conditions. Point charges were calculated by following the ESP procedure with Antechamber at the B3LYP/6-31G** level. The standard protocol for MD simulations was as following: i) energy minimization, ii) solvent equilibration around the solute, and iii) production.

For energy minimization, we used a steepest descent algorithm with 0.01 nm step size until all forces were below 1000 $kJ \cdot mol^{-1} \cdot nm^{-1}$. The equilibration of the solvent consisted of two stages of 1 and 5 ns in steps of 1 fs in which the solute was kept frozen: an initial NVT scheme fixing volume and temperature (298 K), and, subsequently, an NPT scheme where pressure (1 bar) and temperature (298 K) were kept constant. Finally, the production run (NPT scheme) consisted of 20 ns calculations in steps of 1 fs. In all cases, we used a V-rescale thermostat with damping constant of 0.1 ps and a Parrinello-Rahman pressure coupling with damping constant of 5 ps. C−H bonds were constrained with a 4th order LINCS algorithm. The cutoff radius for short-range electrostatic and van der Waals interactions was set to 1.6 nm and we used an order 4 Particle Mesh Ewald for long-range electrostatics.

7.3. Additional results

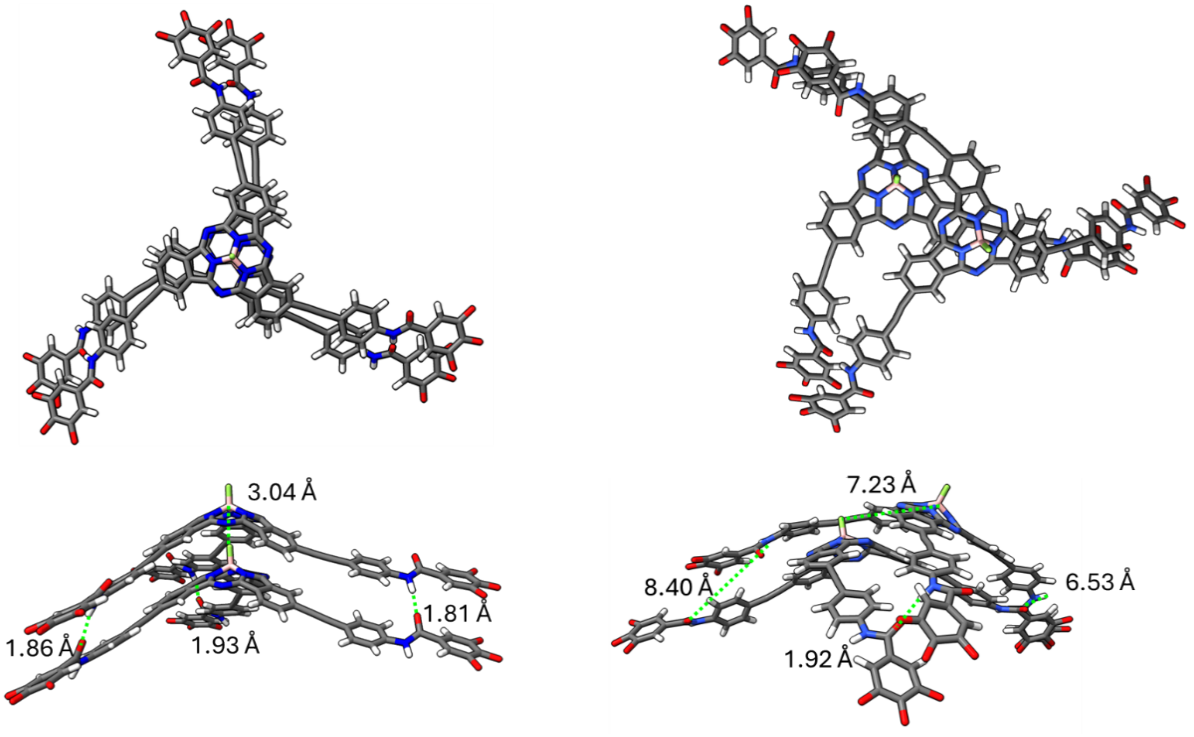

Figure S7.5. Structural models for the columnar (left) and partially dissociated (right) structures found for the $\mathbf{1_2}$-***M*** dimer along the MD trajectory in MCH. Top: top view. Bottom: side view. The values of the F···B and NH···O distances (in Å) are displayed, and the alkyl chains are hidden for better visualization.

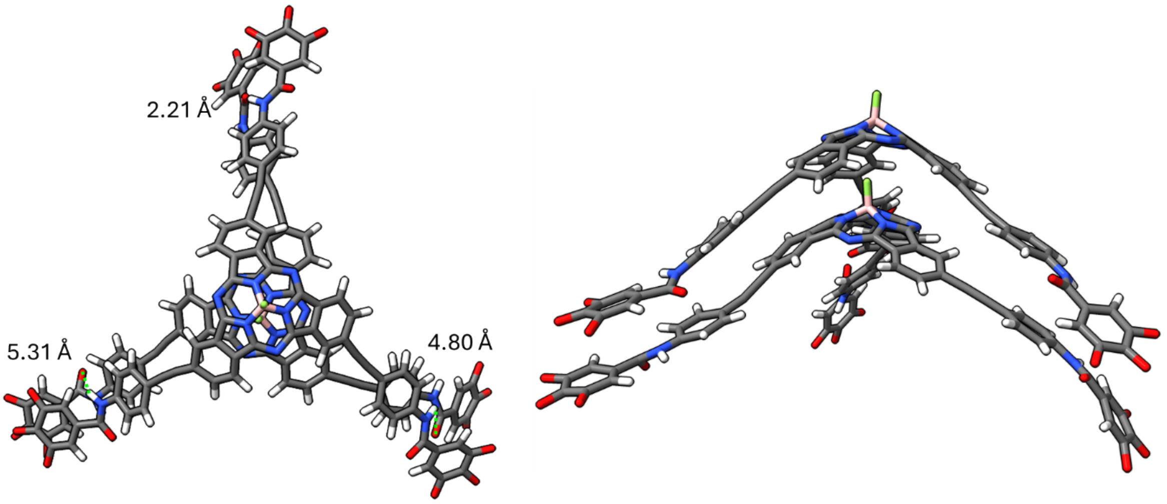

Figure S7.6. Structural snapshot of the $\mathbf{1_{8(2)}}$-***Rac*** central dimer, extracted from the $\mathbf{1_8}$ octamer MD trajectory (left: top view; right: side view), emphasizing the competition between NH···O bonds (top substituent), π-stacking between phenyl rings (right substituent), and π-stacking in addition to a rotation of the amide group (left substituent). The values of the NH···O distances are displayed, and the alkyl chains are hidden for better visualization.

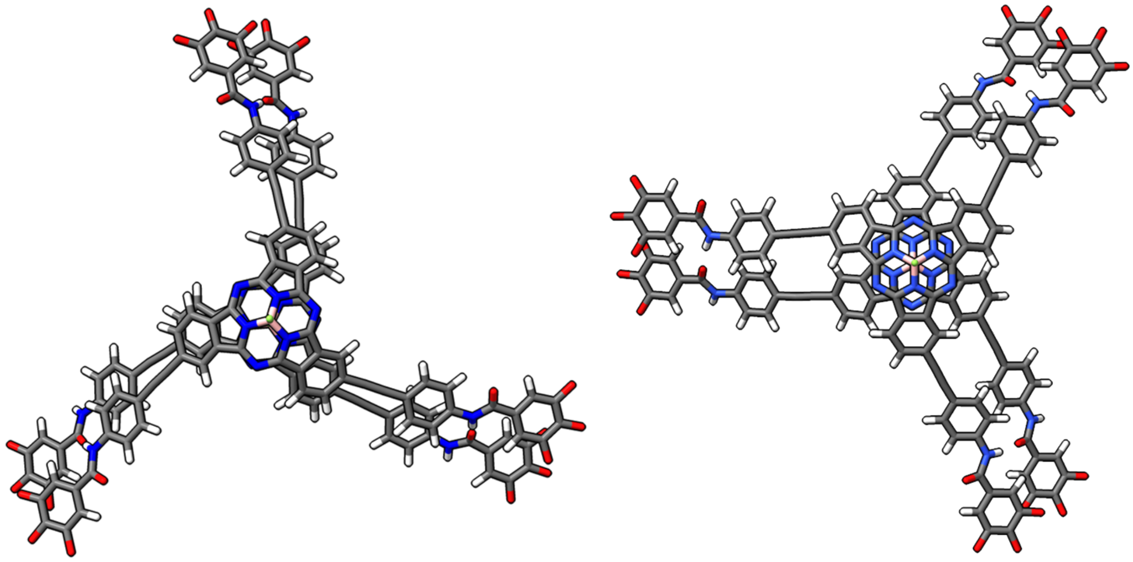

Figure S7.7. Top views of the minimum-energy QMDFF-optimized structures computed in MCH for the columnar structure of homochiral $\mathbf{1}_{8(2)}$-***M*** (left) and heterochiral $\mathbf{1}_{8(2)}$-***Rac*** (right). The most favorable orientation to form strong NH···O bonds between amides is compatible with the π-stacking of the SubPc cores and also of the substituents in **1-*M*** but not in **1-*Rac***, leading to a less stable structure for the latter.

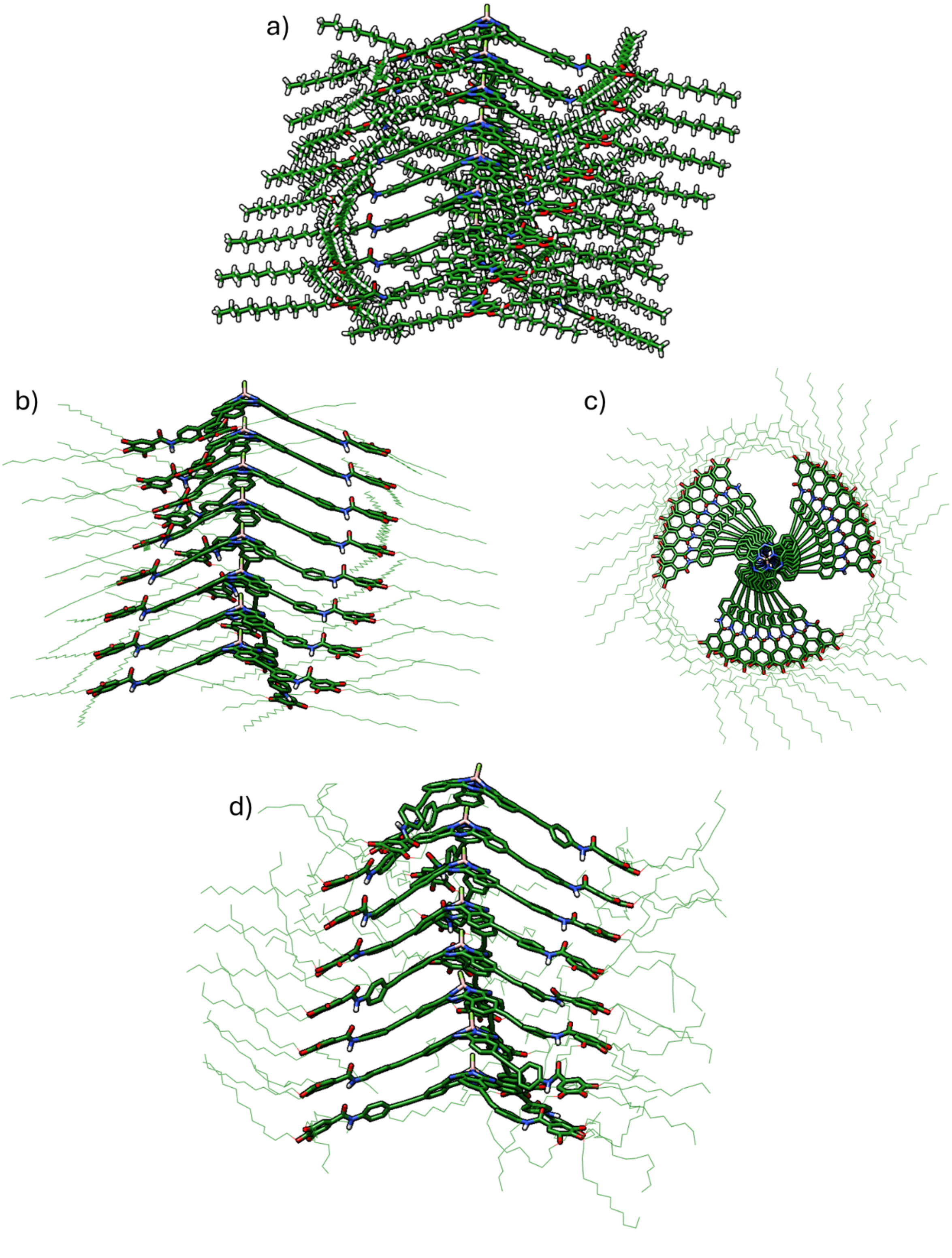

Figure S7.8. a) Minimum-energy QMDFF-optimized structure computed in MCH for the columnar regular self-assembly of the homochiral **1_8-*M*** octamer showing all atoms. Side (b) and top (c) views of the QMDFF-optimized structure of **1_8-*M*** hiding non-H-bonded hydrogens and with lateral alkyl chains as wires for visualization. d) Side view of a structural snapshot extracted from the **1_8-*M*** octamer MD trajectory in MCH.

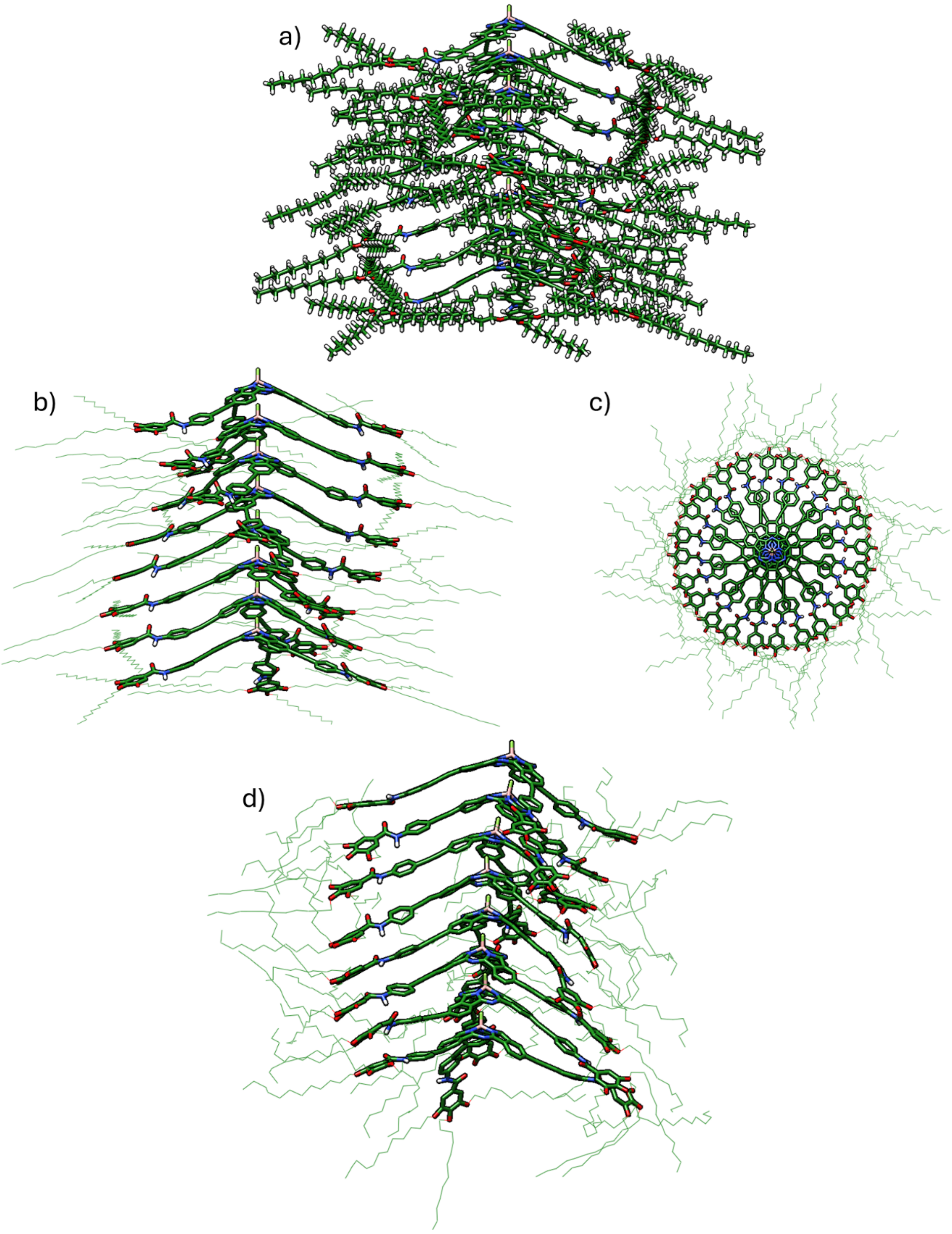

Figure S7.9. a) Minimum-energy QMDFF-optimized structure computed in MCH for the columnar regular self-assembly of the heterochiral $\mathbf{1_8}$-***Rac*** octamer showing all atoms. Side (b) and top (c) views of the QMDFF-optimized structure of $\mathbf{1_8}$-***Rac*** hiding non-H-bonded hydrogens and with lateral alkyl chains as wires for visualization. d) Side view of a structural snapshot extracted from the $\mathbf{1_8}$-***Rac*** octamer MD trajectory in MCH.

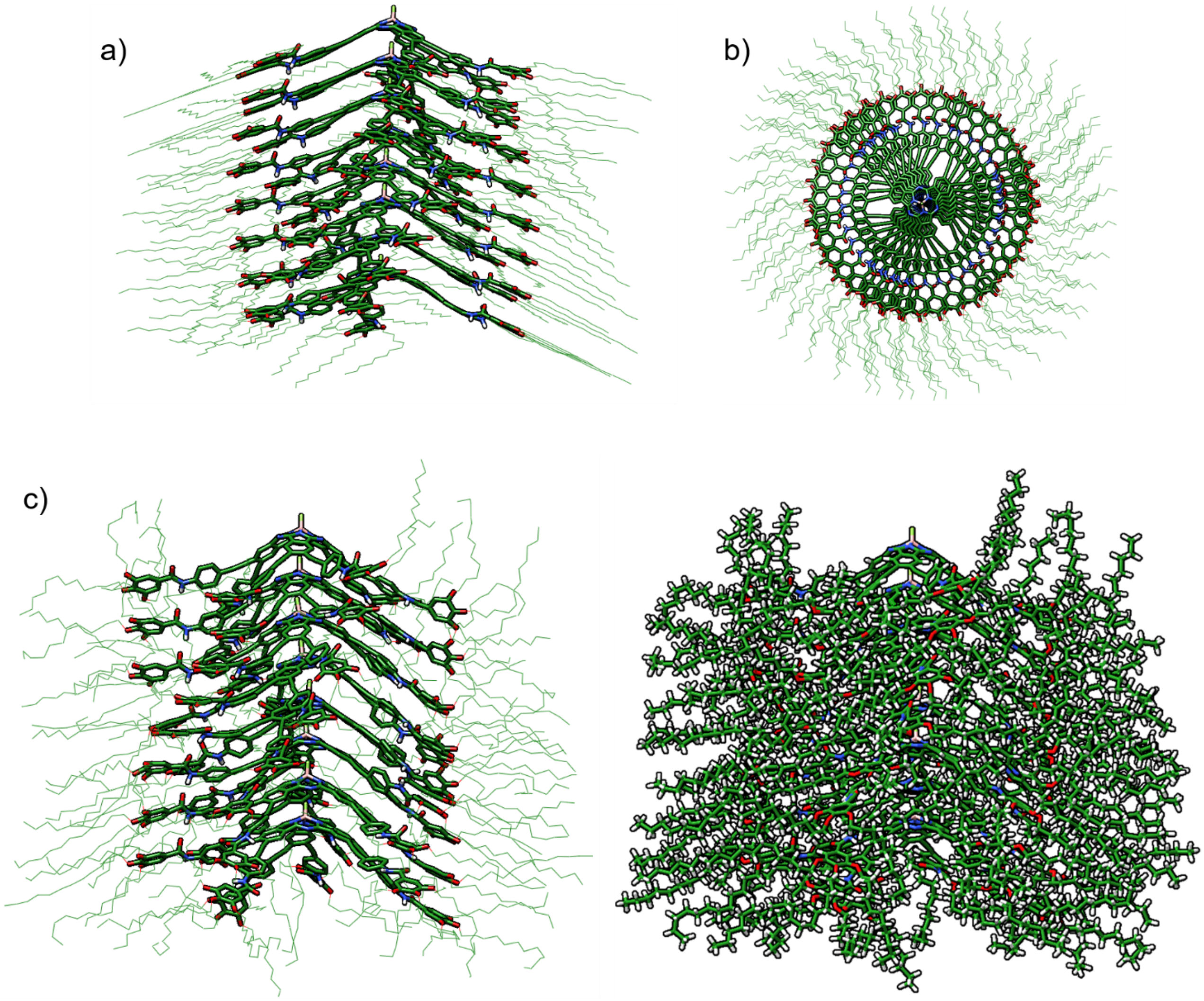

Figure S7.10. Side (a) and top (b) views of the minimum-energy QMDFF-optimized structure computed in MCH for the columnar regular self-assembly of the $\mathbf{2_8}$ octamer hiding non-H-bonded hydrogens and with lateral alkyl chains as wires for visualization. c) Side view of a structural snapshot extracted from the $\mathbf{2_8}$ octamer MD trajectory in MCH.

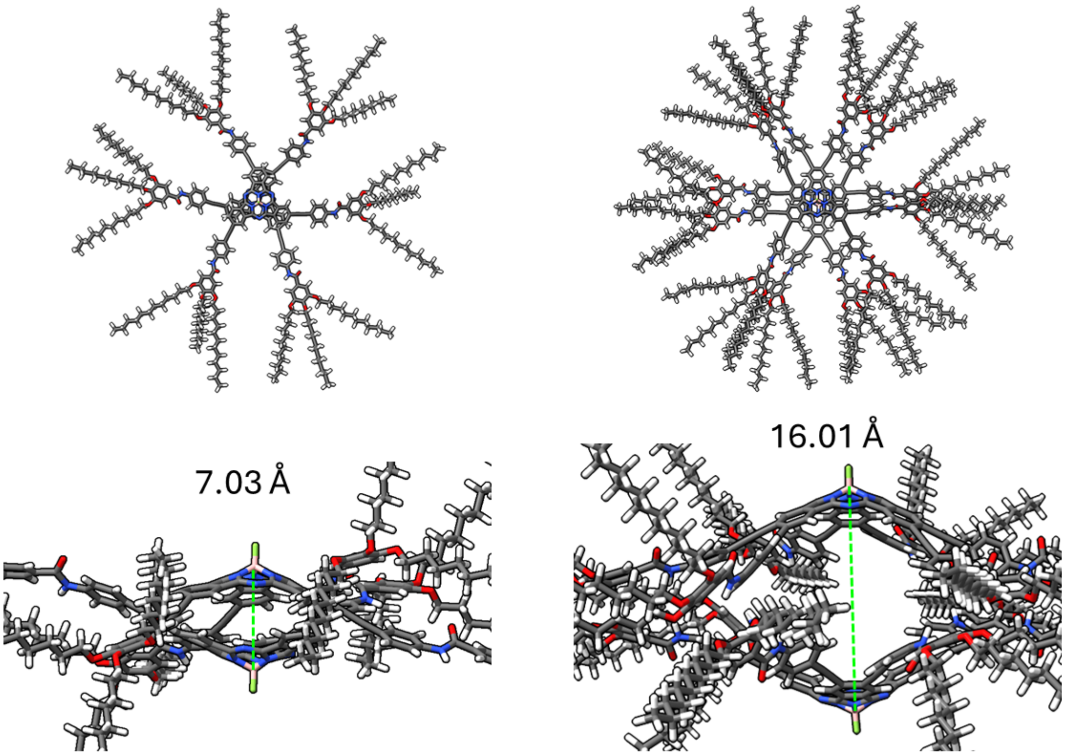

Figure S7.11. Structural models for the optimized tail-to-tail dimers of **1-*M*** (left) and **2** (right) at FF level. Top: top view. Bottom: side view emphasizing the SubPc moieties. The value of the B···B distance is displayed.

References

[1] N. R. Babij, E. O. McCusker, G. T. Whiteker, B. Canturk, N. Choy, L. C. Creemer, C. V. D. Amicis, N. M. Hewlett, P. L. Johnson, J. A. Knobelsdorf, F. Li, B. A. Lorsbach, B. M. Nugent, S. J. Ryan, M. R. Smith, Q. Yang, *Org. Process Res. Dev*. **2016**, *20*, 661–667.

[2] F. Camerel, B. Donnio, T. Dintzer, S. Toffanin, R. Capelli, M. Muccini, R. Ziessel, V. Uni, V. Cnrs-uni, L. Mate, *J. Am. Chem. Soc.* **2009**, *131*, 18177–18185.

[3] J. Labella, G. Lavarda, L. Hernández-López, F. Aguilar-Galindo, S. Díaz-Tendero, J. Lobo-Checa, T. Torres. *J. Am. Chem. Soc*. **2022**, *144*, 16579–16587.

[4] C. G. Claessens, M. J. Vicente-Arana, Tomás Torres, *Chem. Commun*. **2008**, 6378.

[5] H. Li, T. J. Jensen, F. R. Fronczek, M. G. H. Vicente, *J. Med. Chem*. **2008**, *51*, 502–511.

[6] M. Obłoza, Ł. Łapok, J. Solarski, T. Pedzinski, M. Nowakowska. *Chem. Eur. J.* **2018**, *24*, 17080–17090.

[7] a) A. J. Maarkvort, H. M. M. Ten Eikelder, P. J. J. Hilbers, T. F. A. De Greef, E. W. Meijer, *Nat. Commun*. **2011**, *2*, 509. b) H. M. M. Ten Eikelder, A. J. Markwoort, T. F. A. De Greef, P. A. J. Hilbers, *J. Phys. Chem. B*. **2012**, *116*, 5291–5301.

[8] P. A. Korevaar, C. Schaefer, T. F. A. de Greef, E. W. Meijer, *J. Am. Chem. Soc.* **2012**, *134*, 13482–13491.

[9] I. Cacelli, G. Prampolini, *J. Chem. Theory Comput*. **2007**, *3*, 1803–18017.

[10] U. C. Singh, P. A. Kollman. *J. Comp. Chem*. **1984**, *5*, 129–145.

[11] W. L. Jorgensen, J. Tirado-Rives, *J. Am. Chem. Soc*. **1988**, *110*, 1657–1666.

[12] B. Doherty, X. Zhong, S. Gathiaka, B. Li, O. Acevedo, *J. Chem. Theory Comput.* **2017**, *13*, 6131–6145.

[13] M. J. Abraham, T. Murtola, R. Schulz, S. Páll, J. C. Smith, B. Hess, E. Lindahl, *SoftwareX* **2015**, 1–2, 19.

[14] M. Yabe, K. Mori, M. Takeda, *J. Comput. Chem. Jpn Int. Ed*. **2019**, *5*, 2018.
